# Direct GPCR-EGFR interaction enables synergistic membrane-to-nucleus information transfer

**DOI:** 10.1007/s00018-024-05281-5

**Published:** 2024-06-20

**Authors:** Michael Gekle, Robert Eckenstaler, Heike Braun, Abdurrahman Olgac, Dina Robaa, Sigrid Mildenberger, Virginie Dubourg, Barbara Schreier, Wolfgang Sippl, Ralf Benndorf

**Affiliations:** 1 Julius-Bernstein-Institute of Physiology, Martin Luther University Halle-Wittenberg, 06112 Halle (Saale), Germany; 2 Institute of Pharmacy, Department of Clinical Pharmacy and Pharmacotherapy, Martin Luther University Halle-Wittenberg, Halle, Germany; 3 Institute of Pharmacy, Department of Medical Chemistry, Martin Luther University Halle-Wittenberg, Halle, Germany; 4 Institute of Pharmacology and Toxicology, Ruhr-University Bochum, 44780 Bochum, Germany

**Keywords:** Epidermal growth factor receptor, EGFR, Angiotensin II receptor type 1, AT1R, Serum response factor, Heteromerizsation

## Abstract

We addressed the heteromerization of the epidermal growth factor receptor (EGFR) with G-protein coupled receptors (GPCR) on the basis of angiotensin-II-receptor-subtype-1(AT1R)-EGFR interaction as proof-of-concept and show its functional relevance during synergistic nuclear information transfer, beyond ligand-dependent EGFR transactivation. Following in silico modelling, we generated EGFR-interaction deficient AT1R-mutants and compared them to AT1R-wildtype. Receptor interaction was assessed by co-immunoprecipitation (CoIP), Förster resonance energy transfer (FRET) and fluorescence-lifetime imaging microscopy (FLIM). Changes in cell morphology, ERK1/2-phosphorylation (ppERK1/2), serum response factor (SRF)-activation and cFOS protein expression were determined by digital high content microscopy at the single cell level. FRET, FLIM and CoIP confirmed the physical interaction of AT1R-wildtype with EGFR that was strongly reduced for the AT1R-mutants. Responsiveness of cells transfected with AT1R-WT or –mutants to angiotensin II or EGF was similar regarding changes in cell circularity, ppERK1/2 (direct and by ligand-dependent EGFR-transactivation), cFOS-expression and SRF-activity. By contrast, the EGFR-AT1R-synergism regarding these parameters was completely absent for in the interaction-deficient AT1R mutants. The results show that AT1R-EGFR heteromerisation enables AT1R-EGFR-synergism on downstream gene expression regulation, modulating the intensity and the temporal pattern of nuclear AT1R/EGFR-information transfer. Furthermore, remote EGFR transactivation, via ligand release or cytosolic tyrosine kinases, is not sufficient for the complete synergistic control of gene expression.

**Supplementary Information:**

The online version contains supplementary material available at 10.1007/s00018-024-05281-5.

## Introduction

The epidermal growth factor receptor (EGFR) family consists of four tyrosine kinase receptors EGFR (ErbB1), ErbB2, ErbB3 and ErbB4 [1], forming homo- and heterodimers. EGFR controls various signalling modules, thereby affecting transcriptional regulation and finally e.g. cell proliferation, survival, differentiation, migration and matrix homeostasis [2]. In addition to its classical ligands, EGFR is also subject to activation by crosstalk with other receptors, i.e. transactivation. Canonical EGFR-activation as well as EGFR-transactivation are of physiological and pathophysiological relevance, including cell transdifferentiation, proliferation and para-inflammatory dysregulation of tissue homeostasis.

In this context, G protein-coupled receptors (GPCRs) are of especial relevance [3–5]. Four possible mechanisms of GPCR-EGFR crosstalk, leading to enhanced EGFR activity are suggested. (i) GPCRs can activate membrane metalloproteinases, which cleave membrane-anchored EGFR ligands and activate EGFRs of the same (autocrine) or adjacent (paracrine) cells. (ii) GPCRs can activate cytosolic tyrosine kinases that phosphorylate cytosolic EGFR tyrosine residues. (iii) GPCRs may interact directly and physically with EGFRs, forming heterocomplexes [4, 6–10]. This interaction is supposed to induce alterations of the cytoplasmic part of EGFRs resulting in enhanced kinase activity. The evidence level for heteromeric complex (or receptor) formation and their cellular relevance is still limited and in part controversially discussed. (iv) The last and most indirect transactivation, results from GPCR-induced expression of EGFR ligands, leading to EGFR activation with a substantial temporal delay.

A prominent and important example of GPCRs interacting with EGFRs is angiotensin II (AII) type 1 receptor (AT1R) [3, 5, 11]. Pharmacological EGFR inhibition and conditional EGFR knockout models [12–24] showed physiological and pathophysiological relevance of EGFR-AT1R crosstalk in the reno-cardiovascular system in vivo. As reviewed by Forrester et al. [11], a great variety of cell types co-express EGFR and AT1R. These include vascular cells, cardiac cells, renal cells, adipocytes, immune cells as well as cells of the central nervous system. Because virtually every cell type expresses EGFR endogenously, any cell that expresses AT1R co-expresses EGFR and AT1R and is subject to a potential EGFR-AT1R crosstalk and synergy.

EGFR transactivation is supposed to be involved in AT1R-induced effects in the reno-cardiovascular system [11], involving EGFR transactivation via A Disintegrin And Metalloproteinase (ADAM) metalloproteinase domain 17 or cSrc kinase [3, 5, 25, 26]. In the case of ADAM, shedding and binding of Heparin-binding EGF-like growth factor (HB-EGF) activates EGFRs whereas cSrc kinase leads to direct EGFR phosphorylation [2]. Recently, AT1R-EGFR heteromerization (either as complex or receptor) has been proposed in addition [9, 27, 28]. As mentioned above, heteromerization is still discussed controversially and the cellular impact is not clear [10]. Apart from heteromerization with EGFR, heteromerization of AT1R with other GPCRs as well as AT1R-homomerization has been reported [10, 29–35].

Investigations on the mechanistic interaction of two receptors often focussed on cytosolic signalling. By contrast, our understanding regarding the consequences of this interaction in terms of information transfer to the nucleus, transcription regulation and finally the transcriptome itself are mostly unknown. It is often not clear whether receptor interaction leads to (i) a linear nuclear signalling or (ii) parallel signalling with even (iii) synergistic or antagonistic effects. Knowledge concerning these consequences is of major importance because nuclear information transfer affects gene expression with major impact on cell fate.

Recently, we have identified and characterized a synergism of EGFR and AT1R regarding serum response factor (SRF) and activator protein 1 (AP1) activation and SRF target gene expression, like cFOS (part of AP1 dimers), which affects the composition and temporal pattern of transcriptome variation [36] and seems to require a certain spatial interaction of the receptors. Because we could not address the necessity of a direct receptor interaction in our previous study, we investigated the role of a direct EGFR-AT1R-interaction in the cell membrane for this synergistic action of the two receptors in the present study. We focused especially on the information convergence at the level of SRF activity and cFOS-expression (as a measure of AP1 activation) at the single cell level.

Here, we present a proof-of-concept study, showing the potential cellular relevance of GPCR-EGFR heteromerization on the basis of AT1R-EGFR interaction. Following verification of receptor interaction and in silico analysis for the prediction of receptor interaction domains, we generated corresponding AT1R-mutants and confirmed their interaction deficiency with EGFR. Next, we ensured their canonical functionality and finally compared them with wildtype AT1R in synergy tests. Although the canonical signalling remained functional, the synergistic interaction with EGFR was lacking completely for the interaction deficient mutants. Thus, we provide not only evidence for GPCR-EGFR heteromerization but also substantiate the functional cellular relevance beyond signalling.

## Results

### EGFR-AT1R-interaction at the plasma membrane

**Fig. 1:**
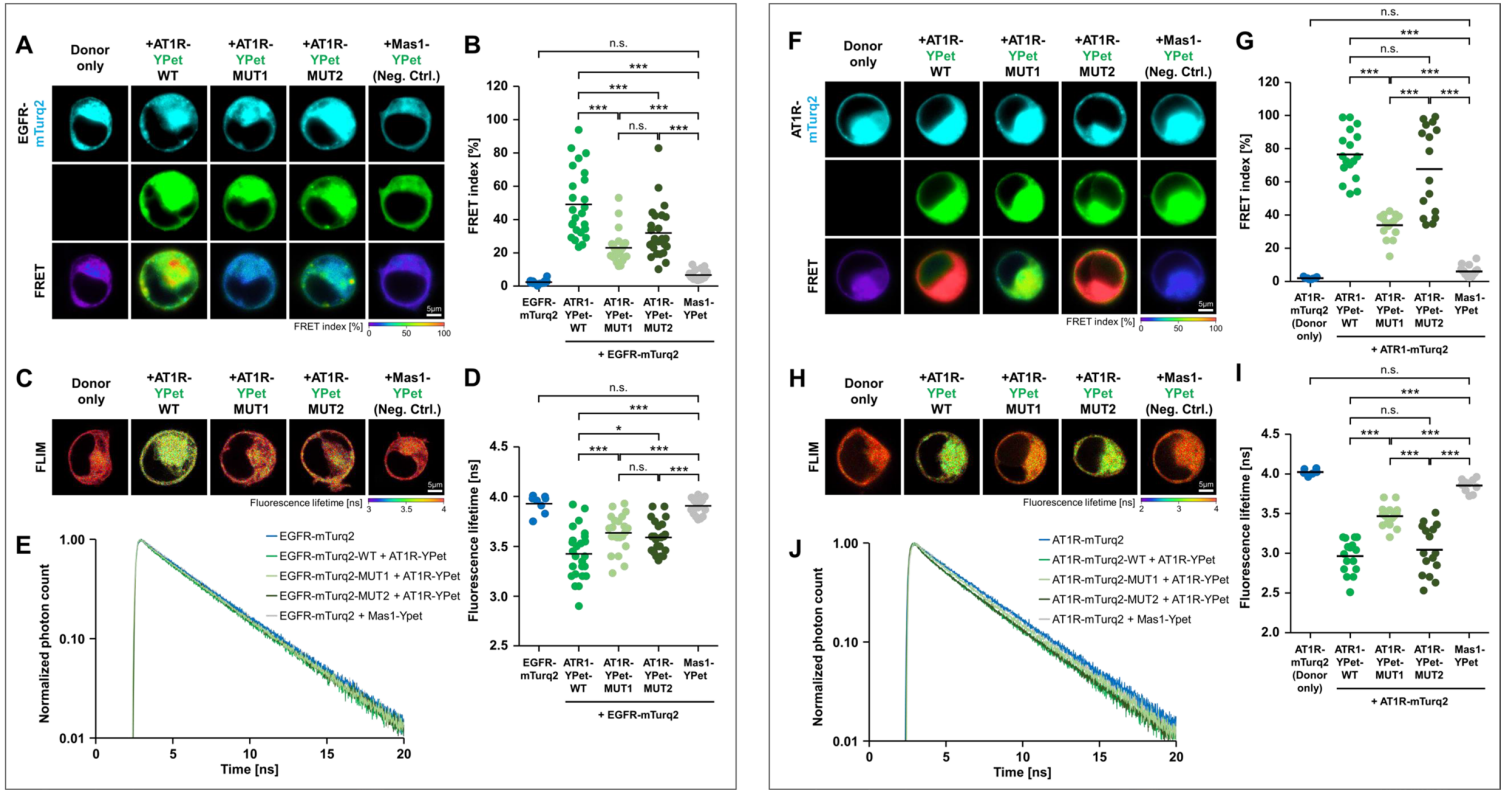
Heteromerization and homomerization of AT1R. **A**–**E** Interaction of EGFR with AT1 receptor. **A** Intensity-based FRET example images obtained for the interaction of EGFR (coupled to mTurqoise2) with AT1 receptor (coupled to YPet) in its wildtype protein sequence (WT) or mutated versions (MUT1, MUT2). Donor only sample (EGFR-mTurquoise2) and EGFR with non-interacting Mas1 receptor served as negative controls. **B** Quantification of FRET indices of plasma membrane regions of the different conditions. A moderate interaction between EGFR and wildtype AT1 receptor could be observed. Mutated AT1 receptor sequences (MUT1, MUT2) significantly reduced this interaction. The addition of YPet-coupled Mas1-receptor did not significantly increase FRET index compared to donor only sample (EGFR-mTurquoise2). **C** Example images of FLIM-based interaction study. **D** Amplitude weighted average fluorescence lifetime of EGFR-mTurquoise2 donor was obtained after 2-exponential fitting of the fluorescence decay in plasma membrane regions of the different conditions. In the presence of wildtype AT1 receptor (coupled to YPet), a FRET-induced significant reduction of fluorescence lifetime was observed. Fluorescence lifetime increased in the presence of the mutant versions of AT1-receptor (MUT1, MUT2) indicating reduced interaction. Non-interacting Mas1-receptor did not significantly alter fluorescence lifetime compared to the donor only sample. **E** Average normalized decay profiles of EGFR-mTurquoise2 fluorescence decay. A faster fluorescence decay due to FRET was observed in case of AT1 receptor interactions. Number of cells = 8–24 from two independent experiments. **F**–**J** Homomerization of AT1 receptor. **F** Intensity-based FRET example images obtained for the homomerization of AT1 receptor (coupled to mTurqoise2) with AT1 receptor (coupled to YPet) monomers in its wildtype protein sequence (WT) and mutated versions (MUT1, MUT2). Donor only sample (AT1R-mTurquoise2) and AT1R with Mas1 receptor served as controls. **G** Quantification of FRET indices obtained for the different conditions. A strong homomerization of AT1 receptors with its wildtype or mutant counterpart (MUT2) could be observed. Counterparts having MUT1 in AT1 receptor sequences significantly reduced homomerization. Compared to donor only sample, no significant change in FRET index was observed in the combination of AT1 receptor and Mas1 receptor. **H** Example images of FLIM-based interaction study. **I** Amplitude weighted average fluorescence lifetime of AT1R-mTurquoise2 donor after 2-exponential fitting of the fluorescence decay in the different conditions. In the presence of wildtype of MUT2-mutated AT1 receptor (coupled to YPet), a significant reduction of donor lifetime (AT1R-mTurq2) due to FRET was observed. Fluorescence lifetime increased in the presence of the MUT1 mutant of AT1-receptor indicating reduced interaction. Mas1-receptor did not significantly reduce lifetime compared to the donor only sample. **J** Average normalized decay profiles of AT1R-mTurquoise2 fluorescence decay. A faster fluorescence decay due to FRET was observed in case of AT1 receptor homomerization. Number of cells = 8–16 from two independent experiments

In order to test an interaction of AT1R and EGFR at the molecular level, we performed immunoprecipitation experiments with N-terminal HA-labelled AT1R and C-terminal EGFP-labelled EGFR expressed in HEK293 cells. Receptors were expressed individually or in combination under basal cell culture conditions. Coimmunoprecipitation (CoIP) and protein detection from cell lysates was performed with anti-EGFP and anti-HA antibodies, respectively. Our findings indicated the formation of AT1R-EGFR heteromers in HEK293 cells (Supplemental Fig. SF01). To further substantiate these conclusions, Förster resonance energy transfer (FRET) and fluorescence lifetime imaging microscopy (FLIM) investigations with receptors C-terminally fused to mTurquoise2 (EGFR) or YPet (AT1R or Mas1, another GPCR to which AII can bind and that was used here as negative control) via a short linker were carried out (Fig. 1A–E). Receptors were expressed individually or in combination and analysed by FRET microscopy or FLIM in living HEK293 cells. Both methods showed FRET upon coexpression of the receptors (AT1R-EGFR), which indicates heteromerization of the receptors in or at the plasma membrane and confirmed the CoIP results.

In order to understand the molecular mechanisms of symmetrical AT1R homodimer formation, molecular models of AT1R were studied based on two of the most common interaction regions of GPCRs, namely (i) TM1-TM2 and TM8 or (ii) TM4 and TM5. The models were generated based on the existing published crystal structure of AT1R, both in active (PDB id 6OS0) and inactive (PDB id 4ZUD) states. Two different interaction scenarios were evaluated in this study (Supplementary Fig. SF02). The first one was conducted with the homodimer crystal structure of CCR4 (PDB id 3OE0), which is in homodimer form on the TM4-TM5 regions (model 1) and the second one was based on the known crystal structure of the AGTR1 homodimer (PDB id 6OS0, model 2). The models indicated that amino acid residues of AT1R in the region of TM5 (S189, I193, L197, I201, L202, L205, F206, model 1) and TM1 and TM3 (Y54, F55, F96, Y99, L100, model 2) might be relevant for the homodimer formation of AT1R. We therefore first generated two AT1R mutant models (S189A, I193A, L197A, I201A, L202A, L205A, F206A (MUT1) and Y54A, F55A, F96A, Y99A, L100A (MUT2)) by homology modelling and analysed the symmetrical homodimerization of these mutants with the AT1R-WT by aligning those structures in their active and inactive states (Supplementary figure SF02). As shown in supplementary figure SF02, we were looking at two forms (active and inactive) of regions known as TM4-TM5. When we aligned these regions on the crystal structure of the CCR4 homodimer, we noticed that they overlap. We generated mutants in this overlapping region (= MUT1, Supplementary Figure SF02, upper panel). Interestingly, this overlapping was absent in the TM1-TM2 and TM8 interface where the second mutant (MUT2) was designed by picking the closest residues at the dimer interface (Supplementary figure SF02, lower panel).

Despite an expression level comparable to that of AT1R-WT and a clear membrane localization of both mutants, AT1R-MUT1 almost completely reversed AT1R homodimer formation and AT1R-MUT2 also tended to have an effect in this direction (Fig. 1F–J). Finally we were able to show by intensity-based FRET and FLIM analyses that both AT1R mutants displayed a significantly reduced heteromer formation with the EGFR (Fig. 1A–E). Surprisingly, we did not observe a pronounced interaction of AT1R with Mas1, although the formation of heterodimers between the two heptahelical receptors has been reported. The reasons are not clear, but could possibly be attributed to different experimental conditions and different cell types studied [37].

### Functional assessment of AT1R mutants

**Fig. 2:**
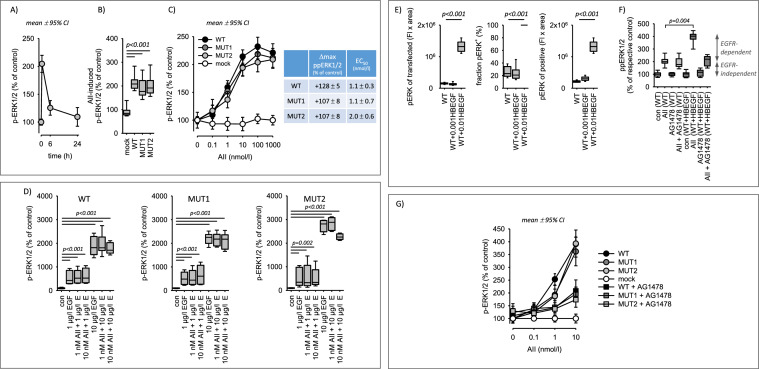
ERK activation in cells expressing AT1R wildtype or AT1R mutants. **A** Angiotensin II (AII, 10 nmol/l) exposure leads to a transient ERK1/2 phosphorylation in cells expressing AT1R (WT). N/n = 6/24. **B** AII-induced (10 nmol/l, 30 min) ERK1/2 phosphorylation is similar in cells transfected with wildtype AT1R (WT) or mutant AT1R (MUT1, MUT2). N/n = 6/24. **C** Concentration-dependent effect of AII on ERK1/2 phosphorylation in cells expressing either wildtype AT1R (WT) or mutant AT1R (MUT1, MUT2). N/n = 6/24. The table shows the maximum effects (Δmax) and potencies (EC_50_) for the different AT1R, obtained by hyperbolic curve fitting. N/n = 6/18. **D** EGF induced similar changes of ERK1/2 phosphorylation after 30 min in cells expressing either wildtype AT1R (WT) or mutant AT1R (MUT1, MUT2). There is no synergism of EGF and AII with respect to ERK1/2 phosphorylation after 30 min. N/n = 6/18. **E** Effect of HB-EGF expression on basal ERK1/2 phosphorylation. Transfection with 0.001 µg plasmid/dish (0.001HBEGF), did not increase basal phosphorylation in contrast to 0.01 µg plasmid/dish (0.01HBEGF). N/n = 6/18. **F** In cells expressing AT1R (WT) and HB-EGF (0.001 µg), AII (10 nmol/l, 30 min) induced a significantly larger and in part AG1478-sensitive (= EGFR-transactivation) ERK1/2 phosphorylation. 1 µmol/l AG1478. **G** AII-induced ERK1/2 phosphorylation by EGFR-transactivation (in cells expressing HB-EGF, 0.001 µg) is similar in cells transfected with wildtype AT1R (WT) or mutant AT1R (MUT1, MUT2). N/n = 6/18. Statistical testing was performed by rank sum test (**B**, **D**, **E**) ANOVA on ranks (**F**)

First, we compared the AII-induced induction of ERK1/2 phosphorylation in cells transfected with WT or mutant AT1R. In HEK293 cells, AT1R-mediated activation of ERK1/2 is independent of EGFR, mainly due to the lack of HB-EGF expression [36]. This allows the controlled and independent investigation of AT1R-induced signalling. HEK293 cells transfected with AT1R responded to AII with a transient and concentration-dependent pERK1/2 phosphorylation (Fig. 2A–C). Transfection of HEK293 cells with mutant AT1R did not affect basal ERK1/2-phosphorylation nor the responsiveness to EGF or phorbol-12-myristate-13-acetate (PMA), used here as a positive control for ERK1/2-activation (Supplementary figure SF03). Concerning AII, responsiveness of mutant AT1R was comparable to WT AT1R (Fig. 2B, C). Merely, MUT2 might respond with a slightly lower potency but has an unaffected maximum response (Fig. 2C). The response to AII with regards to ERK1/2 phosphorylation was composed of a digital (fraction of ppERK1/2-positive cells) and an analogue (intensity of ppERK1/2 signal in ppERK1/2-positive cells) component for all three receptor forms (Supplementary figure SF04). Of note, the digital response displays a higher sensitivity to AII and EGF compared to the analogue response (Supplementary figure SF04). As shown in Fig. 2D, EGF and AII were not additive nor synergistic regarding ERK1/2 phosphorylation. The effect of EGF on pERK1/2 was much stronger compared to AII. We also determined the nuclear fraction of pERK1/2 under the different conditions, because activated ERK1/2 is partially transferred to the nucleus. Supplementary figure SF05 shows that AII, EGF and PMA induced a partial nuclear translocation that was similar for all three AT1R receptors. Furthermore, the data show that AII and EGF did not act additively regarding ppERK1/2 nuclear translocation.

Second, we tested the ability of AT1Rs for ligand-induced EGFR-transactivation. For this purpose, HB-EGF was expressed in HEK293 cells at a level not affecting basal ERK1/2 phosphorylation (Fig. 2E) and the AG1478-senstive (specific EGFR inhibitor) ERK1/2-phosphorylation was determined. HB-EGF expression led to a significantly enhanced AII-induced ERK1/2-phosphorylation that was to a large extent AG1478-sensitive, as shown in Fig. 2F for wild type AT1R-WT. In the absence of HB-EGF, no AG1478-sensitivity was observed. AII-responsiveness and AG1478-sensitivity of cells transfected with mutant AT1R was similar to cells transfected with AT1R-WT (Fig. 2F). Merely, a slightly lower potency with unaffected maximum response may exist.

Thus, the AT1R mutants are functional with respect to ERK1/2 phosphorylation and ligand-dependent EGFR-transactivation, what means that these AT1R effects are independent of receptor heteromer formation.

Next, we assessed the impact of the three different AT1R receptors on morphological cell changes (i.e. circularity) in response to certain external stimuli [38, 39]. We first determined reference values for cellular circularity and size of AT1R-WT transfected HEK293 cells after stimulation with the phorbolester PMA, EGF or AII. Changes in circularity represent a size-independent measure of cell contraction and at the same time may influence information transfer from the membrane into the cell [38]. Supplementary figure SF06A shows the effect of PMA on cellular circularity and size. Both parameters increased non-transiently, although with different kinetics. By contrast, EGF exerted no major effect on the two parameters (Supplementary figure SF06B). AII induced a transient increase in circularity (i.e. a shift to a slightly rounder morphology) of cells transfected with AT1R-WT (Supplementary figure SF06C). The effect on circularity was concentration dependent, with a half-maximum effect at ~ 1 nmol/l. In addition, we performed a subanalysis, measuring the changes in circularity in AT1R-WT transfected cells (determined by RFP expression) with cells showing in addition a positive SRF response (SRF^+^) to AII. Supplementary figure SF06C shows that the effect on cell circularity was stronger in SRF^+^ cells, indicating a certain functional link of the two cellular responses that was not investigated further.

**Fig. 3:**
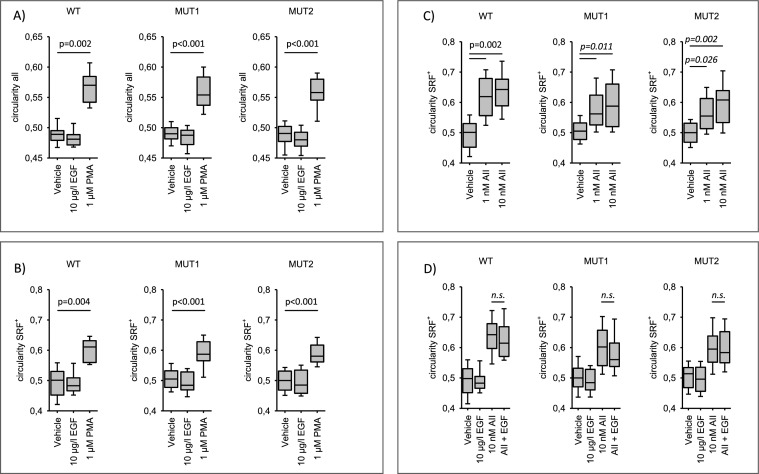
Changes in shape of cells expressing AT1R wildtype or AT1R mutants. **A** and **B** PMA induced similar changes in circularity of cells expressing either wildtype AT1R (WT) or mutant AT1R (MUT1, MUT2). With enhanced circulatity and cell size. N/n = 6/24. **C** Angiotensin II (AII) induced similar changes in circularity of cells expressing either wildtype AT1R (WT) or mutant AT1R (MUT1, MUT2). N/n = 7/42. **D** There is no synergism of AT1R and EGFR activation concerning cell circularity (for comparability the values EGF and AII from panels B and C are replotted). N/n = 6/36. Statistical testing was performed by rank sum test (**A**, **B**, **C**) or ANOVA on rank versus vehicle (**D**). *ns*  not significant

Subsequently, we compared circularity and changes in circularity in cells transfected with either AT1R-WT, AT1R-MUT1 or AT1R-MUT2. As shown in Figs. 3A and B the response to PMA as well as the non-response to EGF were the same for all three receptors. Figure 3C shows the effect of 1 and 10 nmol/l AII on circularity. Again, the response was the same for all three AT1R forms. Finally, we assessed the impact of simultaneous AT1R and EGFR activation on circularity. There was no additive effect (Fig. 3D).

**Fig. 4:**
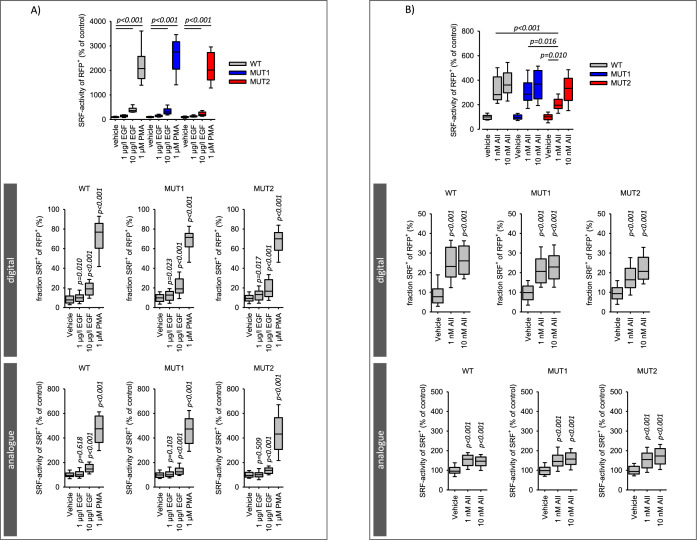
SRF activation in cells expressing AT1R wildtype or AT1R mutants. **A** PMA and EGF-induced SRF activation was not affected by the type of AT1R expressed. PMA and EGF induced similar total, digital and analogue SRF-activation in cells expressing wiltdtype AT1R (WT) or mutated AT1R (MUT1, MUT2). N/n = 10/36. **B** Angiotensin II (AII) induced a similar total, digital and analogue SRF-activation in cells expressing either wildtype AT1R (WT) or mutant AT1R (MUT1, MUT2). Only for 1 nmol/l AII in AT1R-MUT2 expressing cells a difference compared to AT1R-WT was observed. N/n = 11/42. Statistical testing was performed by ANOVA on ranks for total SRF activity and rank sum test versus control for the digital and analogue effects

In a previous study [36] we described and validated the single cell analysis of serum response factor (SRF) activation, including the response mode (digital versus analogue). We showed that HEK293 cells respond to AII only after AT1R transfection, due to the lack of endogenous AT1R expression. Now, we used this experimental system to compare AT1R-WT with AT1R-MUT1 and AT1R-MUT2. Figure 4A shows that transfection with AT1R-MUT1 or AT1R-MUT2 did not alter the responses to PMA or EGF compared to transfection with AT1R-WT, excluding non-specific effects of the mutations. Figure 4B shows the SRF response to AII (1 and 10 nmol/l) in cells transfected with either AT1R-WT, AT1R-MUT1 or AT1R-MUT2. Cells responded to AII in all three cases, showing that all the mutated receptors are functional with respect to single AII application, similar to the results on ERK1/2-phosphorylation, EGFR-transactivation and cell morphology. The only difference we could observe, was a reduced response to 1 nmol/l AII in AT1R-MUT2 transfected cells compared to AT1R-WT or AT1R-MUT1. This difference resulted from a reduced fraction of SRF^+^ cells but not from the analogue response (SRF-activity of SRF^+^ cells).

### Interaction of EGFR with AT1R wildtype and AT1R mutants with respect to SRF activity

**Fig. 5:**
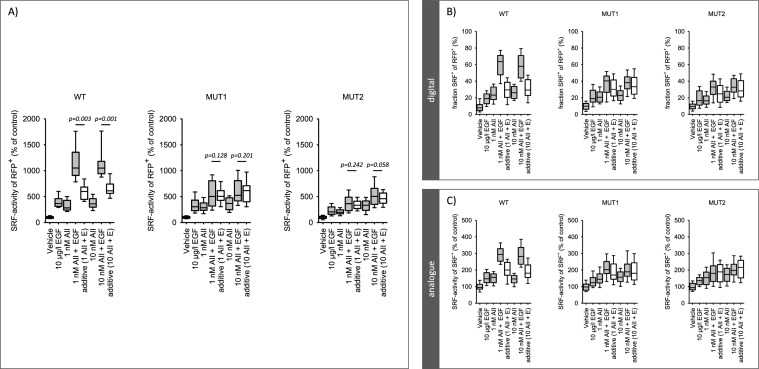
Synergistic SRF activation in cells expressing AT1R wildtype. **A** Simultaneous application of AII and EGF induced a synergistic (over-additive) activation of SRF in cells expressing wildtype AT1R (WT) but not in cells expressing mutated AT1R (MUT1, MUT2) that are deficient concerning physical interaction with EGFR. 10 µg/l EGF. N/n = 10/40. The values for the bars marked “additive” were obtained by adding the effect of EGF and the effect of AII, each applied alone [calculation of additivity: (activity % of control)_EGF_ + (activity % of control)_AII_ −100%]. **B** and **C** AII and EGF induced a synergistic digital (**B**, fraction of SRF-positive cells of transfected cells = RFP-positive) and analogue (**C**, SRF-activity of SRF-positive cells) activation of SRF in cells expressing wildtype AT1R (WT) but not in cells expressing mutated AT1R (MUT1, MUT2) that are deficient concerning physical interaction with EGFR. N/n = 10/40. Statistical testing was performed by ANOVA

Figure 5A and supplementary figure SF07 show the overadditive (i.e. synergistic) effect of EGF and AII (at 1 and 10 nmol/l) on SRF-activity in AT1R-WT-transfected cells, as reported before [36]. When the cells were transfected with AT1R-MUT1 or AT1R-MUT2, the effect of simultaneous application of EGF and AII was additive but no longer synergistic (Fig. 5A). Figure 5B and C show the digital and analogue responses. As for total SRF activity, the EGFR-AT1R synergism was observed only for wild type AT1R but not for the interaction deficient mutants. Thus, the EGFR-AT1R-synergism depends on the direct interaction of the two receptor types.

**Fig. 6:**
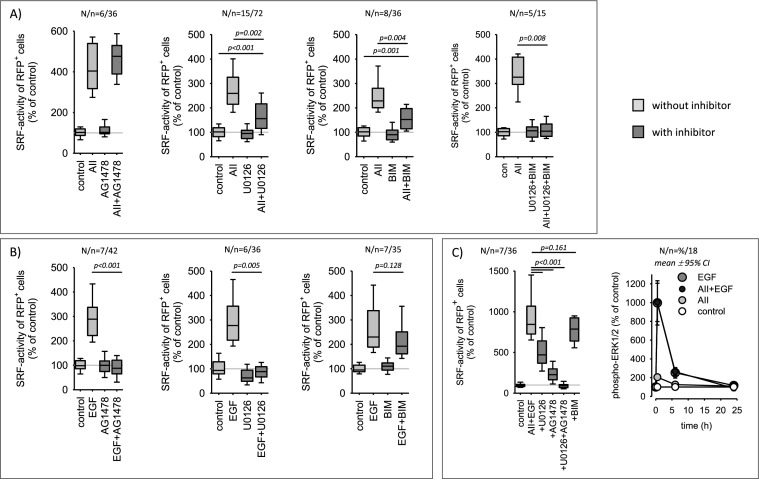
Pharmacological characterization of SRF-activity induction by AII (10 nmol/l), EGF (10 µg/l) or AII + EGF. **A** AII action is insensitive to AG1478 (1 µmol/l, EGFR-inhibition), but senstive to inhibition of the ERK1/2 and the protein kinase C pathway by U0126 (1 µmol/l) or BIM (100 nmol/l), respectively. U0126 + BIM blocked the effect of AII completely. **B** EGF action is blocked completely by AG1478 or U0126 but is insensitive to BIM. **C** The synergistic activation of SRF by simultaneous application on AII and EGF was partially reduced by U0126 or AG1478 and inhibited completely by AG1478 + U0126. This synergism was insensitive to BIM. Concerning ERK1/2-phosphorylation no synergism of AII and EGF was observed. Statistical testing was performed by ANOVA on ranks. Unadjusted p-values versus control are given

As shown in Fig. 6A, AII-induced SRF activation is insensitive to EGFR-inhibition by AG1478 but partially sensitive to inhibition of the ERK1/2 or the protein kinase C pathway by U0126 or BIM, respectively. Simultaneous inhibition of the ERK1/2 and the protein kinase C pathway (U0126 + BIM) prevented AII-induced SRF activation completely. EGF-induced SRF activation was blocked completely by AG1478 or U0126, whereas BIM was not effective (Fig. 6B). AT1R-EGFR synergism regarding SRF-activity, was partially inhibited by U0126 (~ 50% inhibition) and by AG1478 (~ 75% inhibition). The combination of U0126 and AG1478 blocked synergistic SRF activation entirely (Fig. 6C). Concerning ERK1/2-phosphorylation no synergism was observed also after prolonged exposure (Fig. 6C, right panel).

### Synergistic action of EGFR and AT1R with respect to cFOS expression

**Fig. 7:**
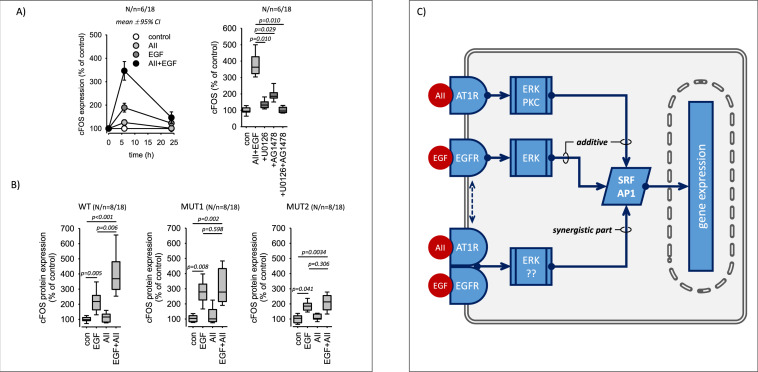
cFOS expression in cells expressing AT1R wildtype or AT1R mutants. **A** Simultaneous application of AII (10 nmol/l) and EGF (10 µg/l) for 6 h induced a synergistic (over-additive) expression of cFOS protein in cells expressing wildtype AT1R (WT). Inhibition of ERK1/2-phosphorylation (1 µmol/l U0126) or EGFR-activity (1 µmol/l AG1478) reduced the induced cFOS expression significantly but not completely. AG1478 + U0126 inhibited completely. **B** Synergistic cFOS induction was not observed in cells expressing mutated AT1R (MUT1, MUT2) that are deficient concerning physical interaction with EGFR. 10 nmol/l AII, 10 µg/l EGF. **C** Scheme of AT1R-EGFR crosstalk with respect to SRF activation and cFOS expression. The upper part shows the additive effect that requires no direct AT1R-EGFR interaction. The lower part represents the synergistic part of the crosstalk that requires direct interaction. Statistical testing was performed by ANOVA on ranks

In our previous study [36] we showed that the AT1R-EGFR synergism translates into the synergistic expression of cFOS, which is a rapidly regulated part of the transcription factor AP1 and also a SRF-target protein. Thus, we investigated whether mutated AT1R exerts similar synergistic effects on cFOS expression as AT1R-WT or not. Figure 7A shows that simultaneous AT1R-WT and EGFR activation enhances the expression of cFOS protein synergistically and transiently, as reported before. cFOS induction was partially inhibited by U0126, although U0126 fully prevented ERK1/2-phosphorylation over the same time period (Supplementary figure SF08), suggesting that its expression in not exclusively regulated via the ERK1/2 signalling pathway. Figure 7B shows that synergistic cFOS-induction occurred in cells expressing AT1R-WT but not in cells expressing the interaction deficient AT1R mutants (see also supplementary figure SF09). These data support the above conclusion of a functionally relevant direct interaction of the two receptors (Fig. 7C).

## Discussion

The relevant functional interaction of AT1R and EGFR—as prime example for GPCR-EGFR interaction—with respect to cytoplasmic signalling has been shown in various studies for several cell types [3, 5, 11]. The most prominent effect of this interaction concerning signalling, is the activation of MAP kinases, followed by transcription factors (e.g. SRF, AP1) that may impact cell differentiation, function and proliferation. Thereby, EGFR is an important signalling hub, enabling the full spectrum of cellular AII actions. While this seems to be of special importance for pathological AII actions, like cell transdifferentiation and parainflammatory dysregulation of tissue homeostasis, leading for example to vascular or renal tubulointerstitial dysfunction and remodelling, there is additional evidence that EGFR is also required for physiological AII actions [3, 5, 11, 12, 22–24, 40].

The underlying mechanisms of this functional receptor interaction include EGFR transactivation [3], either by shedding of membrane bound EGFR-ligands or by EGFR phosphorylation via cytosolic tyrosine kinases of the cSrc family [2, 3, 25]. For these two mechanisms substantial evidence has been provided. Besides this, more direct, physical AT1R-EGFR interaction (heteromerization, either as heteromeric complex or heteromeric receptor) has been proposed [9, 10, 27, 28, 36]. However, the occurrence and functional relevance of AT1R-EGFR heteromerization remained controversial.

Based on the results of our previous study [36], where we identified a functional synergy of AT1R and EGFR concerning SRF and AP1 activation, the expression of target genes and the temporal pattern of transcriptome variation, we tested the contribution of AT1R-EGFR heteromerization to this synergistic nuclear information transfer. Our data show that a close, physical AT1R-EGFR interaction is required for the synergistic, i.e. overadditive, effect on SRF activity and cFOS expression. After in silico prediction of relevant amino acid residues in AT1R for heteromer formation, we generated two AT1R mutants and confirmed their partial inability to interact with EGFR in living cells, in contrast to AT1R-WT. Therewith, we had a tool in hand to investigate whether direct AT1R-EGFR interaction is required for their synergistic effect on transcription control.

Because the responsiveness of cells transfected with either AT1R-WT or AT1R-mutants to AII was similar regarding ERK1/2-phosphorylation, ligand-dependent EGFR-transactivation and cell shape (circularity), we conclude that the AT1R-mutants are in principle functional regarding their canonical signalling and the reduced homomerization capacity has little impact in this regard. We cannot exclude a slight shift in receptor affinity, because ERK1/2-phosphorylation induced by 1 nmol/l angiotensin II as well as circularity alterations were somewhat lower in cells expressing AT1R-mutants compared to AT1R-WT. This was not the case for 10 nmol/l angiotensin II. Non-specific alterations of signalling can be excluded, because AT1R-independent effects induced by PMA or EGF were not affected by the AT1R-mutants.

Concerning SRF, activation of both AT1R-mutants by AII led to a similar total, digital and analogue SFR responses as AT1R-WT at 10 nmol/l angiotensin II, confirming the functionality of the mutants. As for ERK1/2-phosphorylation, the response of cells expressing AT1R-mutant-2 was somewhat smaller at 1 nmol/l but not at 10 nmol/l angiotensin II, compared to AT1R-wildtype. Non-specific alterations of signalling can be excluded, because neither the PMA- nor the EGF-induced activation of SRF was affected by AT1R-mutants. Thus, our experimental setup enabled us to test the hypothesis that a close physical interaction of AT1R and EGFR is required for synergistic SRF activation and cFOS expression.

Cells expressing AT1R-WT showed a significantly overadditive, i.e. synergistic, SRF response (total, digital and analogue) when AT1R and EGFR were activated simultaneously, confirming the data from a previous study [36]. This synergy was completely absent in cells expressing one of the two AT1R-mutants that showed a considerably reduced physical interaction with EGFR (Fig. 1). Because canonical AT1R-signaling of the “heteromerisation-deficient” mutants, regarding ERK1/2-phosphorylation, EGFR-transactivation and SRF activation as well as cell shape changes, by angiotensin II alone, were not impaired, the lack of synergistic AT1R-EGFR effects is explained by the additional close physical AT1R-EGFR interaction. Thus, this heteromerization is a prerequisite for synergistic transcription control.

We also determined the effect of simultaneous AT1R and EGFR activation on cFOS expression, a rapidly regulated component of the AP1 transcription factor and a typical SRF target gene. Again, the receptor synergism was apparent only for AT1R-WT but not for the interaction-deficient AT1R mutants. We conclude that a receptor heterocomplex mediates the synergistic effect of AT1R and EGFR (on top of the additive impact), requiring the close interaction of AT1R and EGFR. SRF and AP1 activation by either of the two receptors are independent of this interaction, as is the mere additive effect (Fig. 7C).

The pathways involved in downstream signalling of the synergistic AT1R-EGFR action are not yet entirely identified. Inhibition of the ERK1/2-pathway by U0126 reduced the induced SRF activity by ~ 50%. The remaining activity does not seem to depend on PKC, because BIM exerted virtually no effect, but it was completely abrogated by the addition of AG1478, an EGFR-inhibitor. As we have shown before, AII-EGF-induced SRF activation is sensitive to latrunculin B, an inhibitor of MRTF-mediated SRF activation and simultaneous application of U0126 + latrunculin B blocked synergistic SRF activation by more than 90% [36]. Thus, AT1R-EGFR synergy seems to be mediated in large part by the ERK1/2 and MRTF pathways that are supposed to be the two major signalling cascades (Rho/actin/MRTF and MAPK/TCF signalling) for SRF activation and cFOS expression. Because SRF and AP1 mediate the expression of a large group of target genes [41, 42] and regulate, for example, switches from a contractile to a proliferative phenotype of vascular smooth muscle cells [43–45], AT1R-EGFR synergism is of importance for the fine tuning of cellular signalling networks, the transcriptome and finally the cell phenotype. Heteromerization seems to change the intensity and the temporal pattern of nuclear AT1R/EGFR-information transfer, e.g. by delaying the termination of information transfer. The molecular mechanisms engaged by AT1R-EGFR heterocomplexes to initiate information transfer via these pathways have to be investigated in future studies.

Regarding the heteromer composition, we cannot exclude the possibility of a larger protein complex that involves scaffolding proteins (i.e. heteromeric complexes), mediating or facilitating the interaction of AT1R and EGFR. At present, it is also not known whether the heterocomplex contains EGFR-dimers that interact with AT1R or even AT1R-dimers that interact with EGFR. In any case, the FRET and FLIM data show that AT1R and EGFR must be in very close proximity for the synergistic action. This conclusion is also supported by data from our previous study [36], where we applied proximity labelling mediated by engineered ascorbic acid peroxidase (APEX). Currently, it is not known whether the interaction extends beyond the cell membrane and takes place at endosomal membranes, as the receptors are known to continue signalling during endocytic retrieval. Investigation of these details and their possible functional relevance will be the topic of future studies.

The present study was performed in HEK-cells since it focused on the comparison on AT1R-WT with two AT1R-mutant receptors in a controlled system. The principle cell physiological relevance of the EGFR-AT1R synergism had been shown in our previous study [36], using different cell types (including A7r5 vascular smooth muscle cells) and an extensive transcriptome analysis. In the present follow-up study we determined a possible contribution of EGFR-AT1R-heteromers to the synergism, a mechanistic aspect which can be addressed in a heterologous expression system.

## Methods

### Cell culture

HEK293 (human embryonic kidney cell line, ATCC Cat# CRL-3216, RRID:CVCL_0063) were obtained from ATCC and cultivated in DMEM/Ham’s F-12 medium (FG 4815, Biochrom, Berlin, Germany), supplemented with 10% fetal calf serum (FCS). Medium was changed to DMEM without FCS prior to addition of stimuli. Transfections were performed with Lipofectamine 2000 according to the manufacturer’s instructions (ThermoFisher Scientific, Dreieich, Germany).

### Plasmid transfection for FRET and FLIM measurements

HEK293T were seeded in a 96-well glass bottom plate (Greiner) at a density of 10,000 cells per well and transfected with plasmids expressing AT1R-mTurquoise2, EGFR-mTurquoise2, MAS1-YPet, AT1R-YPet or interaction reducing mutants of AT1R-YPet. Transfection was performed using the TurboFect reagent (Thermo Fisher Scientific) according to the manufacturer`s recommendations. Imaging experiments were performed one day after transfection.

### Förster resonance energy transfer (FRET)

Live-cell FRET imaging was performed at 37 °C using a Nikon A1R confocal microscope equipped with a 60 × oil immersion objective (plan apo lambda, Nikon, n.a. = 1.4), a PMT detector unit (Nikon, Minato, Japan) and a humidified O_2_/CO_2_ cage incubator (okolab, Ottaviano, Italy) as previously described [46]. Images were acquired and processed using the NIS-Elements FRET module (Nikon). Fluorescence of mTurquoise2-taged constructs (FRET donor) was exited using a 405 nm laser diode (Cube 405-100C, Coherent, Santa Clara, USA) and fluorescence emission was detected in the spectral range of the donor (465–500 nm, DD image) and the acceptor (525–555 nm, DA image), respectively. YPet-tagged constructs (FRET acceptor) were exited using the 514 nm laser line of an argon laser (Melles Griot, Bensheim, Germany) and detected in the spectral range of the acceptor (525–555 nm, AA image). Laser power and detector gain were set in a way to obtain best signal intensities while avoiding oversaturation within the region of interest (cell membranes). Calculation of FRET index was calibrated using donor and acceptor only samples to determine the correction factors for donor crosstalk (α) and the acceptor's direct excitation (β) in the DA image. Images displaying the color-coded FRET index were calculated as intensity of the corrected FRET image normalized by the intensity of the donor image according to the following formula (FRET index = 100% * (DA−αDD−βAA)/DD). FRET values were determined from membranous regions of the cells, only.

### Fluorescence lifetime imaging microscopy (FLIM)

Fluorescence lifetime images were acquired using the FLIM upgrade kit (Picoquant, Berlin, Germany) for the Nikon 1AR confocal laser scanning microscope. Fluorescence of mTurquoise2 was excited using a pulsed laser source (PDL 828 Sepia II, Picoquant) at a wavelength of 444 nm and a repetition rate of 20 MHz. Single photons and their arrival times were detected (PicoHarp300, 483/35 filter, Picoquant) using time correlated single photon counting (TCSPC) method. To avoid pile-up effects, the excitation laser intensity was adjusted for each cell to keep maximum count rate below 2000 kcps. Photons were counted for up to 30 cycles at a capture rate of 1 frame per second. Decay profiles were analysed using SymPhoTime 64 software (Picoquant). Membranous regions of the cells were selected and fitted by employing either one- or two-exponential reconvolution fits using a measured instrument response function (IRF). IRF was measured from fluorescein quenched with saturating concentrations of potassium iodide. In case of FRET, two exponential fitting was the best fitting approach and therefore chosen for direct comparison. In diagrams, the amplitude weighted average lifetime is displayed. For the display of the average decay profiles, photon counts were normalized to the peak value and averaged for all cells measured.

### Co-immunoprecipitation

HEK293T cells were grown for 24 h in 25 cm^2^ culture flasks with a densitiy of 1 × 10^6^ cells in DMEM high-glucose supplemented with 10% FCS, 1% penicillin–streptomycin and 1% GlutaMAX (Thermo Fisher Scientific). pLVX-HA-AGTR1 was generated from pLVX-IRES-Neo (Takara catalogue # 632,181) and pcDNA3.1( +)-HA-hAGTR1 (Bloomsburg University cDNA Resource Center, USA, Catalog Number: #AGTR10TN01). PCR was used to introduce a SpeI restriction site at the N-Terminus of HA-hAGTR1 while keeping the NotI restriction site from pcDNA3.1( +)-HA-hAGTR1. The resulting PCR fragment was digested using SpeI and Not1 and cloned into pLVX-IRES-Neo. For expression of human influenza hemagglutinin (HA)-tagged AT1R as well as EGFP-tagged EGFR, transfection of the plasmids pLVX-HA-AGTR1 (Takara bio, San Jose, CA, USA) and pEGFP-EGFR (addgene #32751) alone or in combination was performed by mixing the respective plasmid DNA (2 µg) with 2 M CaCl_2_, water, and 2 × HBS at pH 7.07 and adding the mixture dropwise to the cell culture flasks. After 20 h, medium was changed once. Two days after transfection, proteins were harvested. For this, cells were lysed using a pre-made lysis buffer (Cell Signaling Technology, Danvers, MA, USA) containing 20 mmol/L Tris/HCl, pH 7.5, 1 mmol/L Na_2_EDTA, 1 mmol/L EGTA, 150 mmol/L NaCl, 1% Triton X-100, 2.5 mmol/L Na_4_P_2_O_7_, 1 mmol/L b-glycerophosphate, 1 mM Na_3_VO_4_, and 1 μg/ml leupeptin and a premade protease and phosphatase inhibitors single use cocktail (Thermo Fisher Scientific) as previously described [47]. The lysates were subsequently centrifuged for 5 min, 4 °C, at 1500×*g* to remove cell debris. Receptor-receptor interaction was subsequently determined by Western Blot analyses (description see below).

Co-immunoprecipitation of EGFR-EGFP or HA-AT1R fusion proteins was carried out utilizing the Immunoprecipitation Kit Dynabeads (Thermo Fisher Scientific, Waltham, MA, USA). First, the co-IP bead complex was prepared with 5 µg of the monoclonal GFP antibody JL-8 from Takara bio (order number 632381) or the human hemagglutinin (HA) antibody from Sigma-Aldrich (St. Louis, Missouri, USA; order number H3663). Each protein lysate (1 mg) was added to the HA antibody/Dynabead complex and incubated overnight. The bound proteins were eluted and prepared for Western blot analyses using 2 µl of the respective eluate.

Equal amounts of eluate (2 µl/lane) were separated by SDS-PAGE and transferred to a nitrocellulose membrane. Membranes were then incubated with primary antibody solution (anti-HA tag (clone C29F4), 1:1000, 5% BSA, Cell signaling technologies. Bound antibodies were detected by peroxidase-conjugated secondary antibodies and the ECL system (Amersham Bioscience, Amersham, UK).

### In silico modeling

The inactive state of AT1R was derived from PDB ID: 4ZUD, crystallized in monomer form, while the active state was sourced from PDB ID: 6OS0. The protonation states were determined at pH 7.4 using the *Protein Preparation Wizard* in *Schrodinger Biologics Suite 2023-3*, employing the OPLS4 force field. AT1R homodimer modeling leveraged the structure of the CCR4 homodimer (PDB ID: 3OE0) as a foundation, facilitating the generation of molecular systems representing both inactive and active states of the TM4-TM5 interacting interface. For modeling the inactive state of the TM1-TM2 and TM8 interacting interface, the AT1R homodimer crystal structure (PDB ID: 6OS0), which is present in an active state, was employed. The structural models were generated using *Protein Structure Alignment* tool accessed via *Maestro*.

Due to the clashing observed in the inactive state of TM4-TM5, we proceeded to generate two mutation models, focusing on the active state. This was done using the *Residue Scanning Tool* in *Maestro*, following the alignment steps for both homodimers. Mutation model 1 (MUT1) features alterations including S189A, I193A, L197A, I201A, L202A, L205A, and F206A, while mutation model 2 (MUT2) involves mutations Y54A, F55A, F96A, Y99A, and L100A. The creation of these models leveraged homology modeling approaches and incorporated a relaxation step (with an RMSD of 0.3 Å), allowing for the concurrent mutation of the chosen residues.

### Single cell reporter gene analysis by digital high content microscopy

We assessed activity of the transcription factor SRF by reporter gene assays. Changes in the expression of the reporter gene is a measure for transcription factor activation. Thus, we measured reporter gene activity under different conditions, calculated the changes versus control and denominated these values transcription factor activation (e.g. SRF activation). A detailed description of data acquisition and analysis with exemplary images is given in supplementary methods file. With this approach only signal from transfected cells are recorded, preventing confounding effects from non-transfected cells. Reporter for SRE (sequence GGATGTCCATATTAGGA) transcription factor was purchased from Qiagen, Hilden, Germany. We used the Cignal™ System (http://www.sabiosciences.com/reporterassays.php) with Monster-green fluorescent protein (MGFP) as reporter. The respective transfection control was red fluorescent protein (RFP) under the control of a constitutive CMV promoter. After transfection with Polyfect (Qiagen, Hilden, Germany) cells were incubated as described in the figure legends and reporter activity was determined as recommended by the manufacturer by digital fluorescence microscopy (Cytation 3, BioTek, Bad Friedrichshall, Germany or the PerkinElmer Operetta CLS™ high content screening system). To determine the cellular responses, first transfected cells were identified according to their red fluorescence (Ex 586/15 nm; Em 647/57 nm; DM 605 nm; LED 590 nm) and their number, mean fluorescence intensity, area, circularity and integral fluorescence intensity determined. Cell identification and determination of the parameters was performed with the Gen5 2.09 software (BioTek, Bad Friedrichshall, Germany). For this purpose, the object recognition parameters (background fluorescence, threshold fluorescence change, rolling ball diameter, object minimum and maximum size, light exposure time, light intensity, gain of image acquisition) were determined during three independent training experiments and subsequently applied to all experiments, making them comparable. Second, the mean green fluorescence intensity (Ex 469/35 nm; Em 525/39; DM 497 nm; LED 465 nm) of the red cells as well as the integral green fluorescence of red cells was determined, using the same routine as for red cells. Finally, red cells that were also green were identified and their number, mean fluorescence intensity, area, circularity and integral green fluorescence integral. The change in fraction of red cells (= transfected cells that could respond) that show a green signal (= active SRF) corresponds to the digital response (switching on of previously inactive cells). The change in green fluorescent intensity of green cells corresponds to the analogue response (enhancing the activity of already activated cells). The overall response to a stimulus is the change in green fluorescence (= SRF activity) of all red cells. This overall response results from the changes in digital and the analogue component (Δoverall = Δdigital x Δanalogue).

### Morphological analysis

Cell circularity and cell area, as a surrogate for cell size, were determined from the RFP fluorescence images obtained by digital high content microscopy. Both parameters were calculated by the Gen5 3.11 software. First, cell area (A) and perimeter (P) were measured. Then, circularity (C) was calculated as C = 4 × π × A/P^2^. Theoretically, circularity can range from 0 to 1, representing perfectly linear to completely circular morphology, respectively. An increase in circularity results from cell dedifferentiation or contraction.

### In-Cell-ELISA: single cell immunofluorescence imaging by digital microscopy

To investigate the expression of putative SRF-target genes or the phosphorylation of pERK1/2 in transfected cells only, cells were transfected with pEGFP in addition to AT1R, followed by stimulation as indicated. A detailed description of data acquisition and analysis with exemplary images is given in the supplementary methods file. With this approach only signals from transfected cells are recorded, preventing confounding effects from non-transfected cells, by contrast to immunoblotting aproaches. The approach was validated in our previous study [36] and combines the advantages of flexible transient transfection with the analysis of transfected cells only. After cell fixation with 4% formaldehyde for 24 h at 4 °C, cells were permeabilized (0.1% Triton X-100 in TBS; 37 mg/l Na-orthovanadate) and non-specific antibody binding was blocked using 5% donkey serum in permeabilization buffer. Primary antibodies from Cell Signaling Technologies, Frankfurt, Germany (phospho-ERK1/2 #9101, RRID:AB_331646, 1:1000; cFOS, # 2250, RRID:AB_2247211, GAPDH #2118, RRID:AB_561053, 1:1000) were diluted in 1% BSA in permeabilization buffer and incubated overnight at 4 °C. Donkey anti-rabbit AlexaFluor568 secondary antibody (#A10042, Invitrogen Life Technologies, Darmstadt, Germany) was then diluted 1:500 in 1% BSA in permeabilization buffer and incubated for 1 h in the dark at room temperature. Nuclei were stained by diluting DAPI in PBS at 1 µg/ml and applied for 10 min in the dark at room temperature. Digital microscopy was performed using a 20 × objective. Subsequently the images were analysed with the Gene 5 3.11 software (BioTek, Bad Friedrichshall, Germany) and in-build routines after adjusting the necessary parameters (background, threshold, object size, rolling ball size). The sequence of single cell analysis for was the following: 1. Identify transfected cells by green fluorescence (= EGFP fluorescence) red. 2. Determine cell number, mean cell area, mean fluorescence intensity 3. Determine mean red fluorescence (= protein of interest marked by AlexaFluor568-labelled antibody. 4. Identify transfected cells positive for the protein of interest. 5. Determine number of transfected cells positive for the protein of interest. 6. Determine the intensity of the protein of interest (red fluorescent level) of cells positive for the protein of interest. The sequence of single nucleus analysis for was the following: 1. Identify nuclei by DAPI fluorescence. 2. Identify the subpopulation of nuclei of transfected cells by green fluorescence (= EGFP fluorescence) red. 3. Determine nuclei number, mean nuclear area, mean fluorescence intensity 4. Determine mean red fluorescence (= protein of interest marked by AlexaFluor568-labelled antibody. 5. Identify nuclei of transfected cells positive for the protein of interest. 6. Determine number of nuclei of transfected cells positive for the protein of interest. 7. Determine the intensity of the protein of interest (red fluorescent level) of nuclei from transfected cells positive for the protein of interest.

### Materials

The following plasmids were used: pDsRed2 (#632,406, Clontech, Mountain View, CA; 3.6 ng/cm^2^); pEGFR-C1 (#6084-1, Clontech Laboratories now Takara Bio USA, Göteborg, Sweden; 3.6 ng/cm^2^); pCMV6-XL4-AT1R (SC 108918, Origene, Rockville, MD; 36 ng/cm^2^); pCMV6-XL4-AT1R-MUT1 (obtained by site-directed mutagenesis from SC 108918, Origene, Rockville, MD; 36 ng/cm^2^) and pCMV6-XL4-AT1R-MUT2 (obtained by site-directed mutagenesis from SC 108918, Origene, Rockville, MD; 36 ng/cm^2^), pLVX-HA-AGTR1 (Takara bio, San Jose, CA, USA), pEGFP-EGFR (addgene #32751), pN1-AT1R-YPet (Takara bio, San Jose, CA, USA), pN1-AT1R-YPet-MUT1 (Takara bio, San Jose, CA, USA), pN1-AT1R-YPet-MUT2 (Takara bio, San Jose, CA, USA), pN1-EGFR-mTurquoise2 (Takara bio, San Jose, CA, USA), pN1-AT1R-YPet (Takara bio, San Jose, CA, USA). All cloning procedures regarding the generation of pN1-AT1R-YPet, pN1-EGFR-mTurquoise2 and pN1-MAS1-YPet as well as sequencing confirmation of these steps were carried out by Synbio Technologies (Monmouth Junction, NJ). Site-directed mutagenesis to generate two AT1R mutants AT1R-MUT1 (S189A, I193A, L197A, I201A, L202A, L205A, F206A; S026251-02-K319510), AT1R-MUT2 (Y54A, F55A, F96A, Y99A, L100A; S026251-01-K319394), AT1R-YPet-MUT1 (S189A, I193A, L197A, I201A, L202A, L205A, F206A; S020606-3) and AT1R-YPet-MUT2 (Y54A, F55A, F96A, Y99A, L100A; S020606-1) as well as sequencing confirmation of the mutations was performed by Synbio Technologies (Monmouth Junction, NJ). Unless stated otherwise all materials were purchased from Sigma, Munich, Germany.

### Statistics

ANOVA or Kruskal–Wallis ANOVA on ranks followed by post hoc testing (e.g. Holm-Sidak or Dunn method), Student´s T-Test or Mann–Whitney rank sum test were used as applicable according to pre-test data analysis by SigmaPlot 12.5 (Systat Software, Inc., San Jose, CA) or STATplus (AnalystSoft Inc., Brandon, GB). A p-value < 0.05 was considered significant. Biometrical planning was performed with α = 0.05 and β = 0.8. Experiments on reporter gene expression, ERK1/2-phosphorylation, cFOS-expression and cell morphology were performed with five cells passages or more with 3 or more individually treated cell culture wells in each experiment. The numbers are given as N/n, where N represents the number of passages and n the number of individually treated cell culture wells. The number of passages was used for statistical testing. In each well the transfected cells (identified either by RFP or EGFP) were analysed on a single cell basis and the mean value of all cells analysed in one well used for further evaluation. Thus, the value of each individually treated cell culture well results from several transfected cells (in the range of 300–400 transfected cells per well). For more details see supplementary methods SM01–SM16. The box plots show the median, 10th, 25th, 75th, and 90th percentiles as vertical boxes with error bars. Line plots are presented as mean ± 95% confidence intervals. FRET and FLIM were determined in two independent experiments with 4 or more independent biological replicates in each experiment.

### Supplementary Information

Below is the link to the electronic supplementary material.

Supplementary file1 (PDF 1003 KB)

Supplementary file2 (PDF 4285 KB)

## Data Availability

All data generated or analysed during this study are included in this published article and its supplementary information files.

## Abbreviations

ADAM: A Disintegrin and metalloproteinase
AII: Angiotensin II
AP1: Activator protein 1
AT1R: Angiotensin II subtype 1 receptor
BSA: Bovine serum albumin
cFos: Fos proto-oncogene, AP-1 transcription factor subunit
cSRC: Cellular SRC proto-oncogene, non-receptor tyrosine kinase
DAPI: 4′,6-Diamidin-2-phenylindol
DMEM: Dulbecco's Modified Eagle Medium
EGF: Epidermal growth factor
EGFR: Epidermal growth factor receptor
ErbB: Erythroblastic leukemia viral oncogene
ERK1/2: Extracellular signal-regulated kinases 1/2
FCS: Fetal calf serum
GAPDH: Glyceraldehyde-3-phosphate dehydrogenase
GFP: Green fluorescent protein
GPCR: G-protein coupled receptor
Grb2: Growth factor receptor-bound protein 2
MAPK: Mitogen-activated protein kinase
MEK1/2: Mitogen-activated protein kinase kinase 1/2
MRTF: Myocardin-related transcription factor
PKC: Protein kinase C
PMA: Phorbol-12-myristat-13-acetat
SRE: Serum response element
SRF: Serum response factor
TBS: Tris-buffered saline
TCF: Ternary Complex Factor
