# Supplementary material for: Direct GPCR-EGFR interaction enables synergistic membrane-to-nucleus information transfer
Source: Cell Mol Life Sci. 2024 Jun 20;81(1):272. doi: 10.1007/s00018-024-05281-5 (PMC11335197; doi:10.1007/s00018-024-05281-5)

Supplementary figures (results)

Supplementary figure SF01:

Interaction of EGFR with AT1 receptor.

Co-immunoprecipitation analysis of HA-tagged AT1R and EGFP-tagged EGFR in HEK293 cells. Band 1 (control lysate of untransfected cells) and band 2 (lysate from EGFR-EGFP-expressing HEK293) show IP controls. Band 3 shows the IP result from HEK293 cells that coexpressed HA-AT1R and EGFR-EGFP.

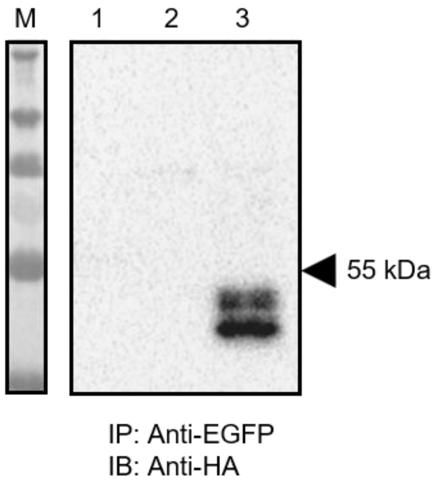

Supplementary figure SF02.

Visualization of the homodimer model 1.

A) Superimposition of the symmetrical AT1R homodimer models on TM4-TM5 interaction region for both active and inactive structures. The red color indicates the inactive and the green color indicates the active form of the structure. The arrow indicates the clashing region on the inactive state. B) Snake representation of the mutated residues (highlighted in yellow), the figure was generated by using GPCRDB (DOI: 10.1093/nar/gkx1109). C) Targeted residues for mutation are represented in yellow, their alanine forms are represented in cyan on the active form of the structure (green).

Visualization of the homodimer model 2.

A) Superimposition of the symmetrical AT1R homodimer models on TM1-TM2 and TM8 interaction region for both active and inactive structures. The red color indicates the inactive and the green color indicates the active form of the structure. B) Snake representation of the mutated residues (highlighted in yellow), the figure was generated by using GPCRDB. C) Targeted residues for mutation are represented in yellow, their alanine forms are represented in cyan on the active form of the structure (green).

Visualization of the homodimer model 1.

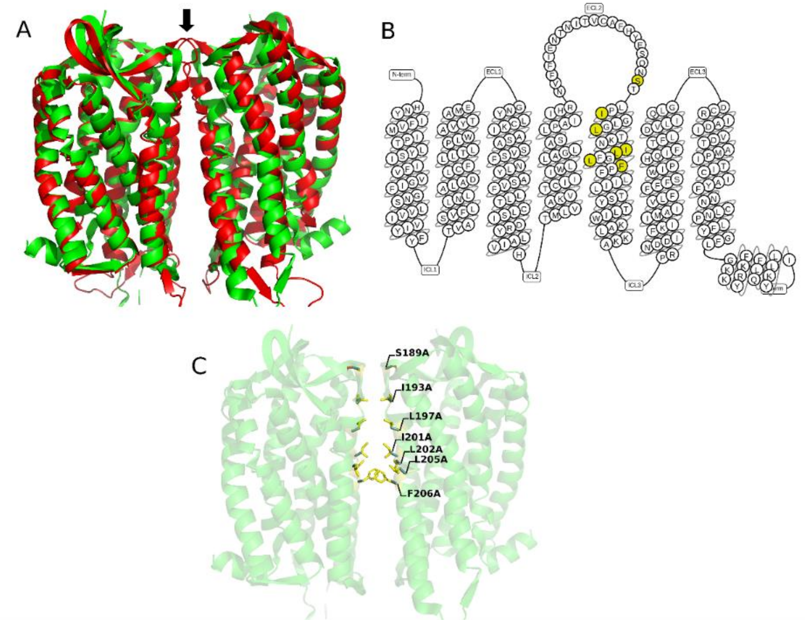

Visualization of the homodimer model 2.

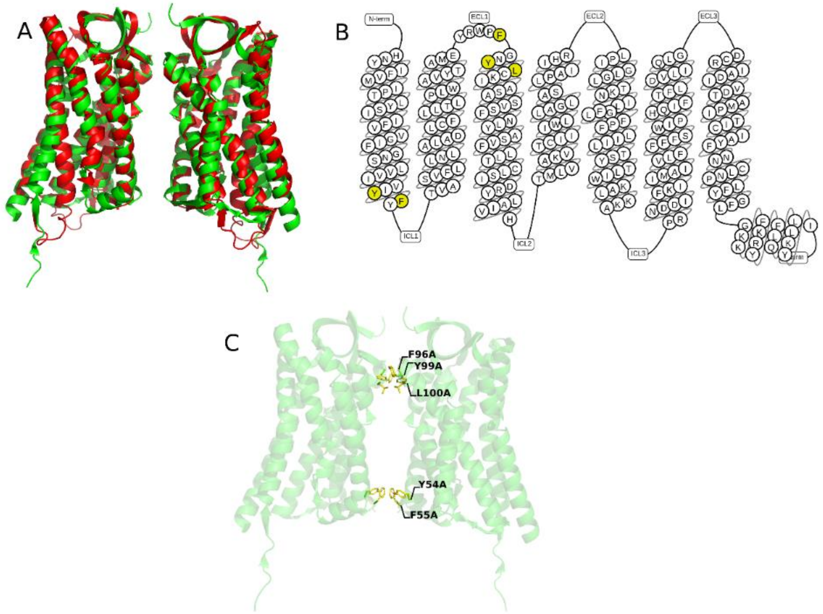

Supplementary figure SF03.

A) Basal ERK1/2-phosphorylation is not different between cells transfected with wild type AT1R (WT) or with mutant AT1R (MUT1, MUT2). N/n = 6/18.

B) ERK1/2-phosphorylation induced by EGF (1 and 10 µg/l) or PMA (1 µmol/l) is not different between cells transfected with wild type AT1R (WT) or with mutant AT1R (MUT1, MUT2). N/n = 8/24. Cell were incubated for 30 minutes. Statistical testing was performed by ANOVA. Unadjusted p-values versus control are given.

A)

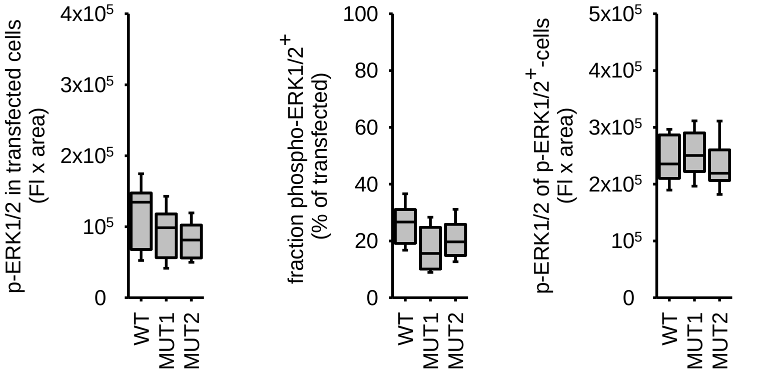

B)

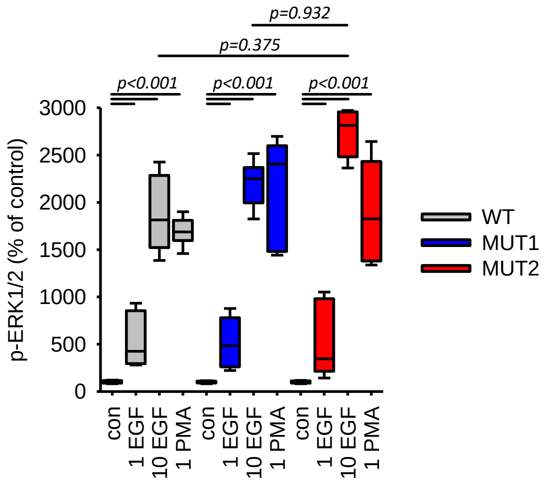

Supplementary figure SF04.

A) Effect of AII, EGF and PMA (1 µmol/l) on the fraction of phospho-ERK1/2-positve cells (= digital response) in cells transfected either with wild type AT1R (WT) or mutant AT1R (MUT1, MUT2). Unadjusted p-values versus control are given.

B) Effect of AII, EGF and PMA (1 µmol/l) on the phospho-ERK1/2-level in phospho-ERK1/2-positve cells (= analogue response) in cells transfected either with wild type AT1R (WT) or mutant AT1R (MUT1, MUT2).

Cell were incubated for 30 minutes. Statistical testing was performed by rank sum test versus control. Unadjusted p-values versus control are given. N/n = 7/20.

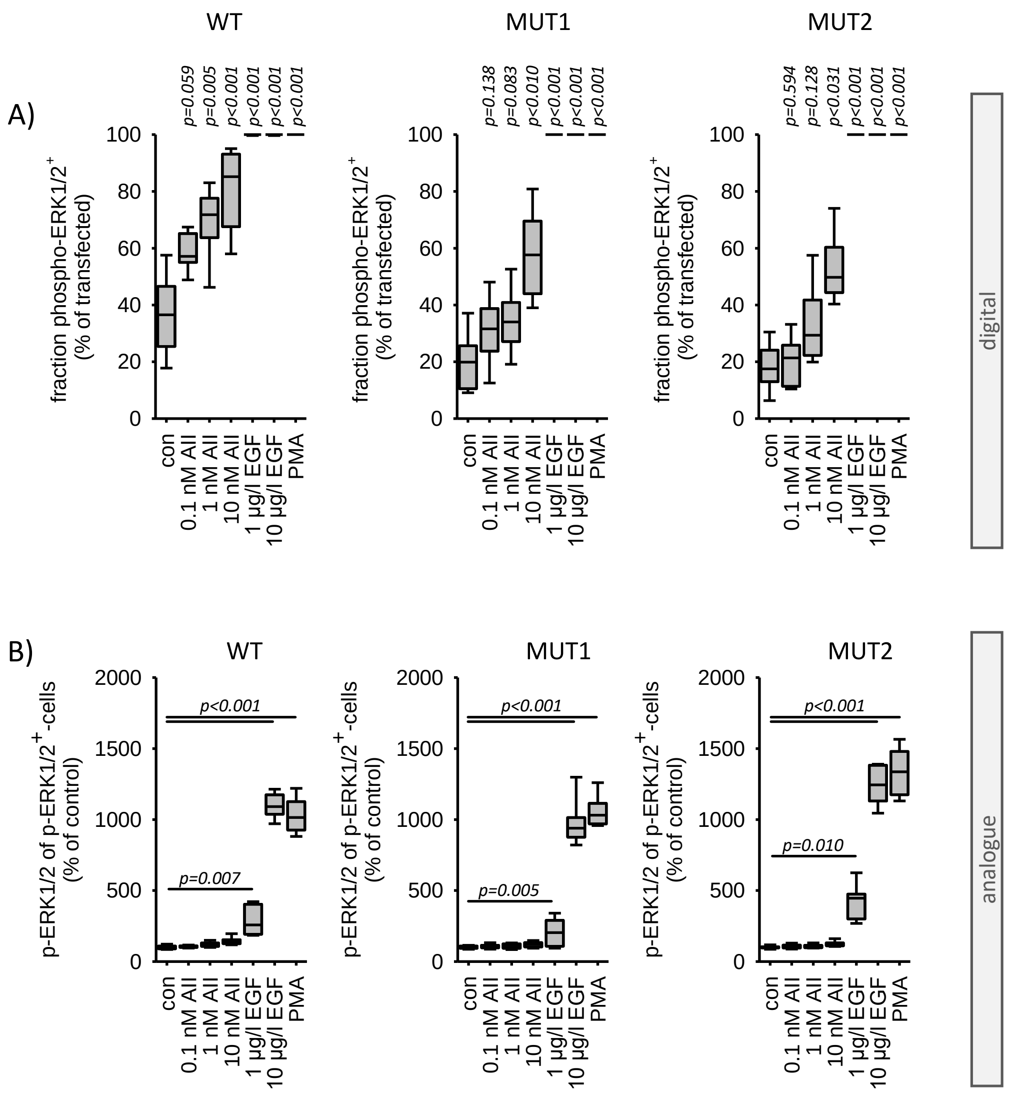

Supplementary figure SF05.

Effect of AII, EGF and PMA (1 µmol/l) on the nuclear fraction of phospho-ERK1/2 relative to total phospho-ERK1/2 (= nuclear translocation) in cells transfected either with wild type AT1R (WT) or mutant AT1R (MUT1, MUT2).

Cell were incubated for 30 minutes. Statistical testing was performed by rank sum test versus control. Unadjusted p-values versus control are given. N/n = 7/20.

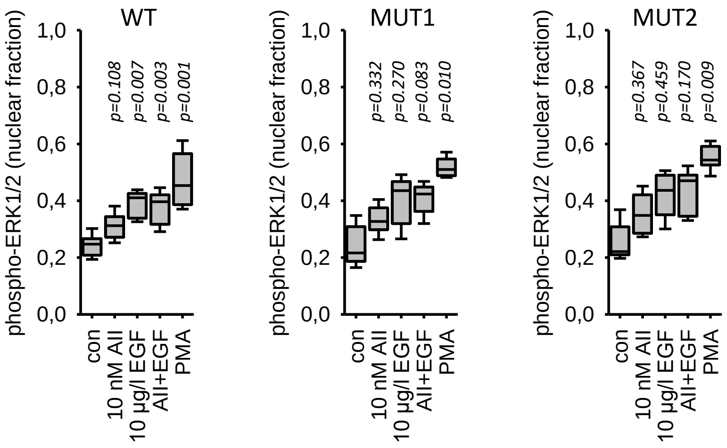

Supplementary figure SF06.

A) PMA induces a rapid and sustained change in cell mporphology with enhanced circulatity and cell size. N/n = 6/36.

B) EGF exerts no major effect on cell morphology. N/n = 6/36.

C) Activation of the wildtype AT1R leads to a transient increase in circularity but only to minor changes in cell size. N/n = 6/36. The EC_{50} values are within the physiological range for AT1R activation. Circularity of SRF^+ cells was higher compared to all transfected cells. Inhibition of EGFR (1 µmol/l AG1478) did not prevent AII-induced changes in circularity.

Statistical testing was performed by rank sum test versus time = 0h (A; C upper left panel) or ANOVA (C, lower right panel). Unadjusted p-values versus control are given.

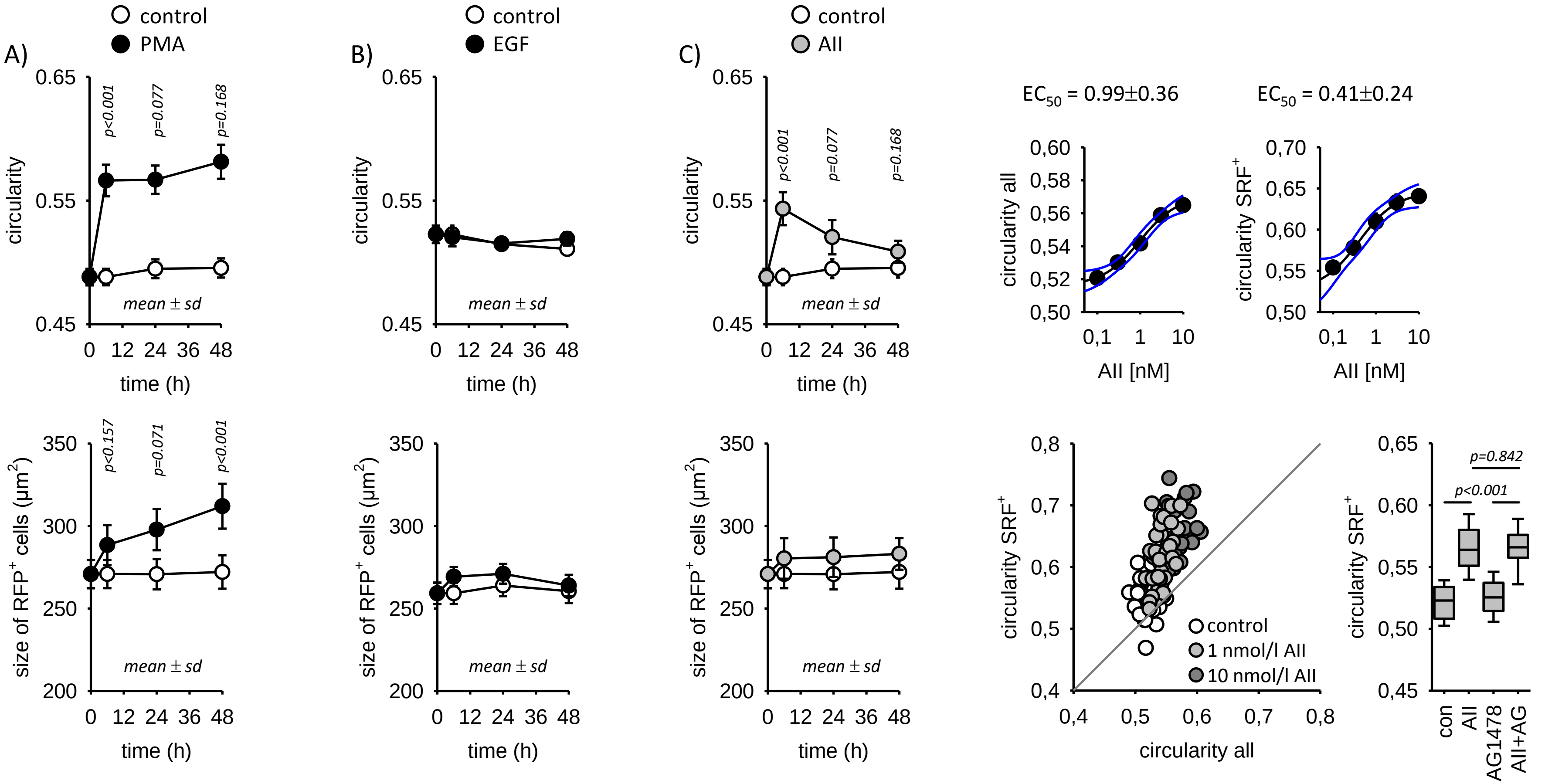

Supplementary figure SF07.

Time-course of SRF-activation in cells tranfected with AT1R wild type. 1 µmol/l PMA. 10 µg/l EGF. 1 nmol/l AII. RFP^+ (RFP-positive cells) identifies transfected cells and enables the analysis of transfected cells only. N/n = 6/30. Statistical testing was performed by ANOVA. Unadjusted p-values versus control are given.

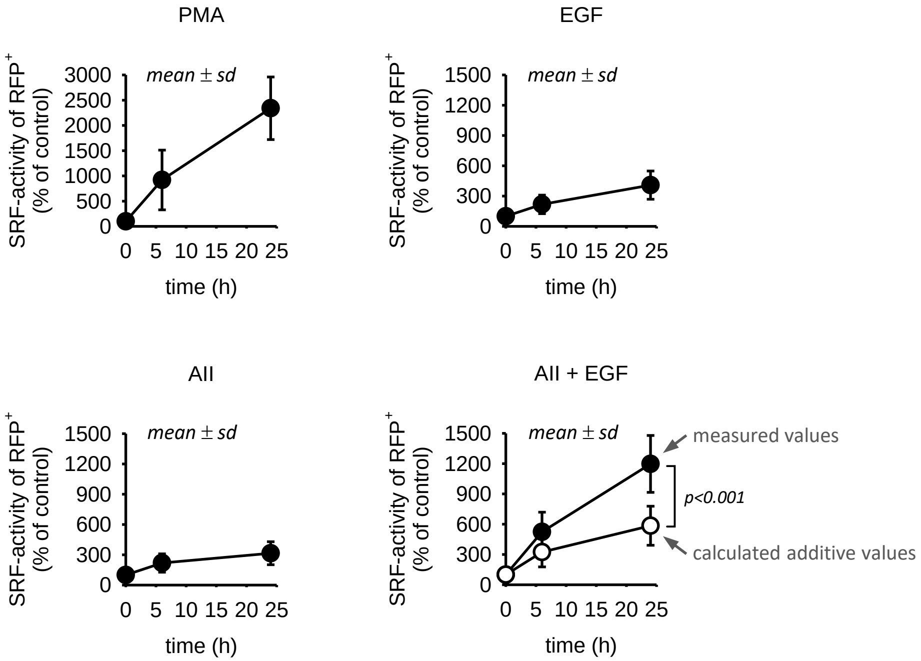

Supplementary figure SF08.

ERK1/2 phosphorylation induced by exposure to 10 nmol/l AII + 10 µg/l EGF for 6 hours was completly blocked by 1 µmol/l U0126. ppERK1/2 = phospho-pERK1/2.

N/n = 5/15. Statistical testing was performed by ANOVA. Unadjusted p-values versus control are given.

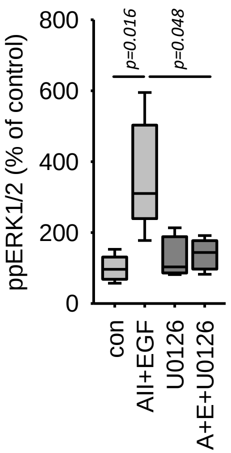

Supplementary figure SF09.

Comparison of the cFOS-level after 6h incubation with AII+EGF. cFOS-levels are expressed as net integral fluorescence in the TR (texas red) channel of tranfected cells (identified in the green channel) and were calculated as [mean TR-fluorescence – background fluorescence] × cell area (µm²).

N/n = 4/12. Statistical testing was performed by ANOVA. Unadjusted p-values versus control are given.

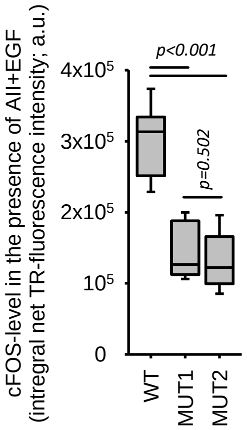

Supplement: Supplementary file 1 — Supplementary file1 (PDF 1003 KB) [file 18_2024_5281_MOESM1_ESM.pdf]
