# Supplementary material for: Direct GPCR-EGFR interaction enables synergistic membrane-to-nucleus information transfer
Source: Cell Mol Life Sci. 2024 Jun 20;81(1):272. doi: 10.1007/s00018-024-05281-5 (PMC11335197; doi:10.1007/s00018-024-05281-5)

Supplementary methods SM01: Basic workflow of digital microscopy applied in reporter assay or in-cell-ELISA (part 1)

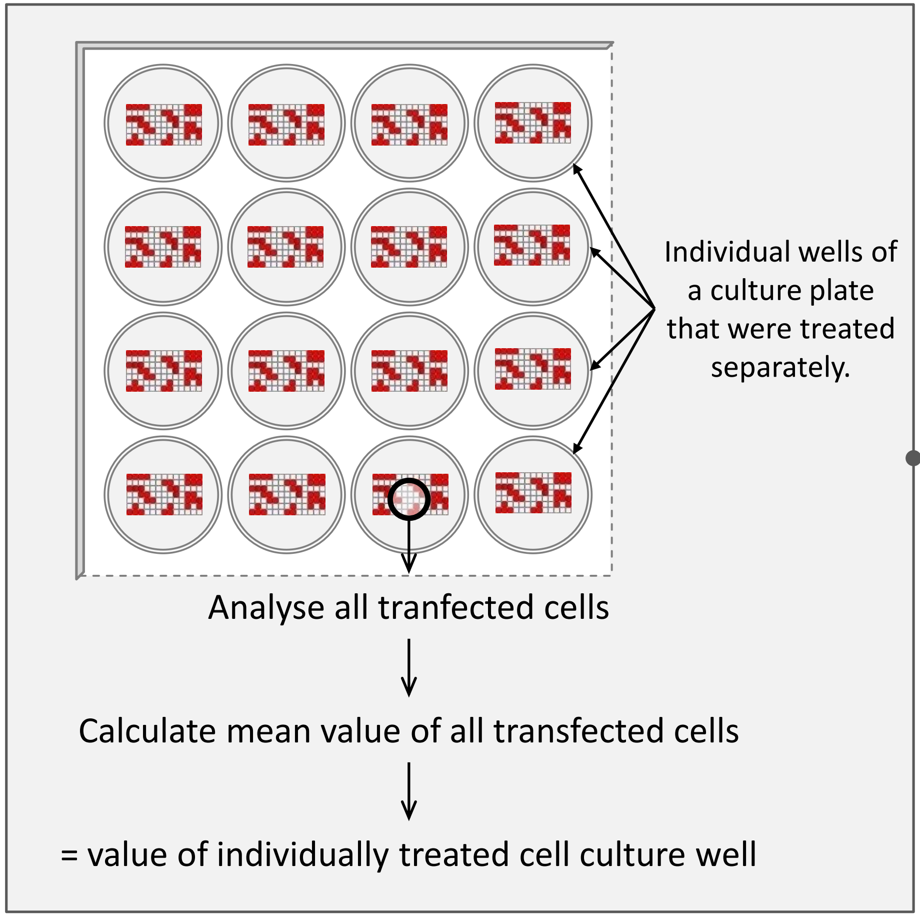

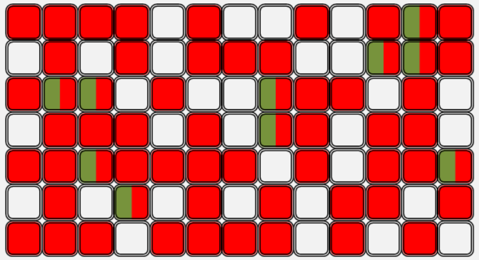

biological effect = response probabilty × response amplitude

↓ digital regulation ↓ analogue regulation

parameter	process
number of red cells / area	transfection, protein synthesis
red fluorescence / cell	protein synthesis
size of red fluorescent cells	hyper-/hypotrophy
number of green cells / area	digital SRF-activity
green fluorescence / cell	analogue SRF-activity
size of green fluorescent cells	hyper-/hypotrophy

For the current study:

Total of ~2500 wells with 3-4 images with ~300 cells

~ 2,4 × 10^6 cell measurements were obtained and used for the analyses

Supplementary methods SM02: Basic workflow of digital microscopy applied in reporter assay or in-cell-ELISA (part 2)

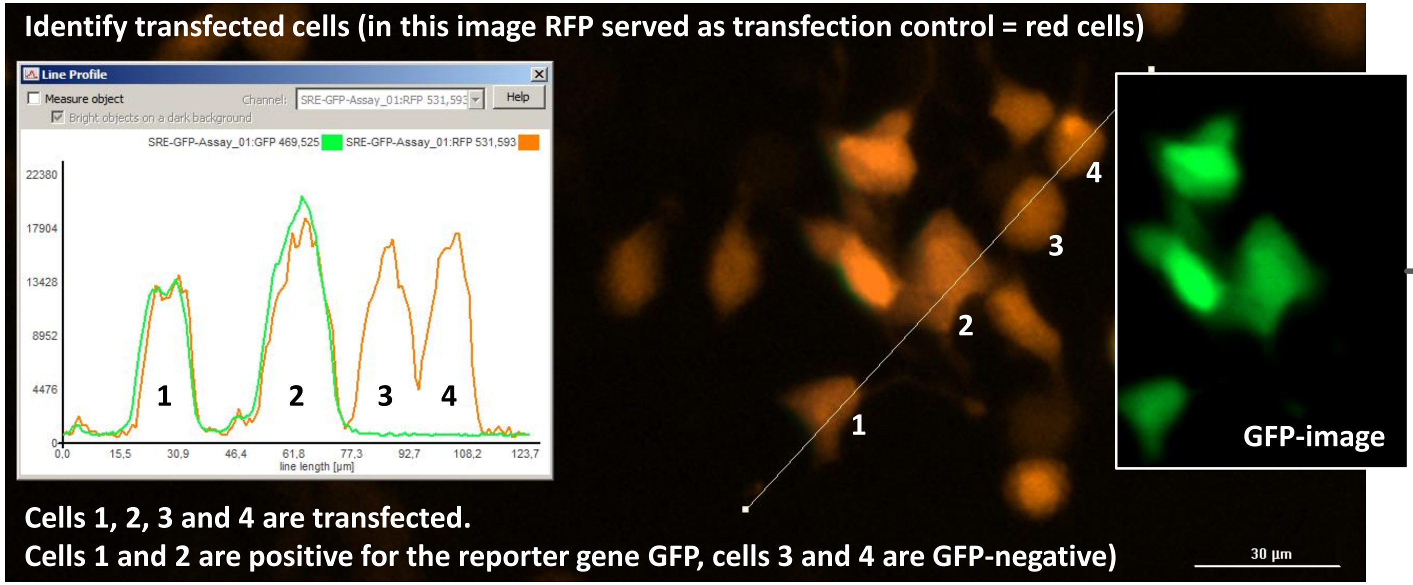

1. Identify transfected cells.
2. Measure signal intensity integral of interest in transfected cells: Either the reporter GFP or an endogenous protein marked by a specific primary and an Alexa Fluor 568 labelled secondary antibody.

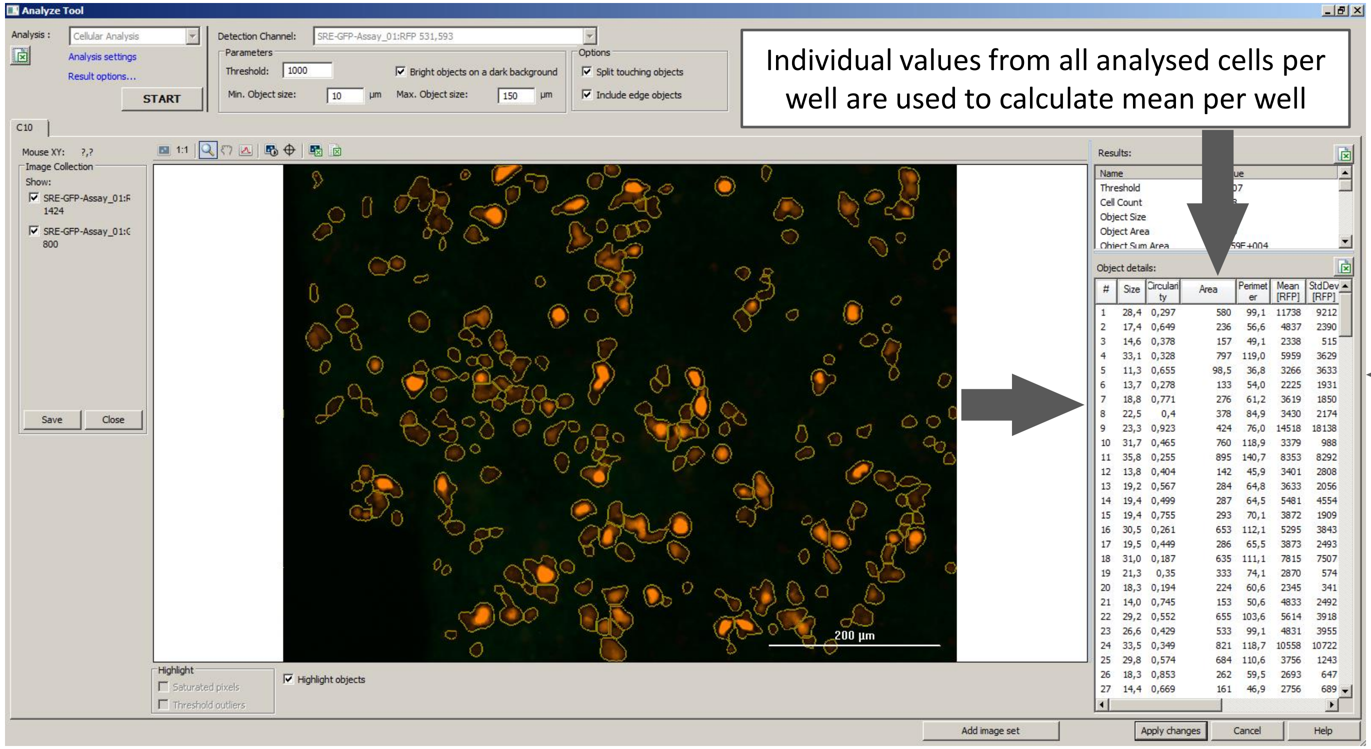

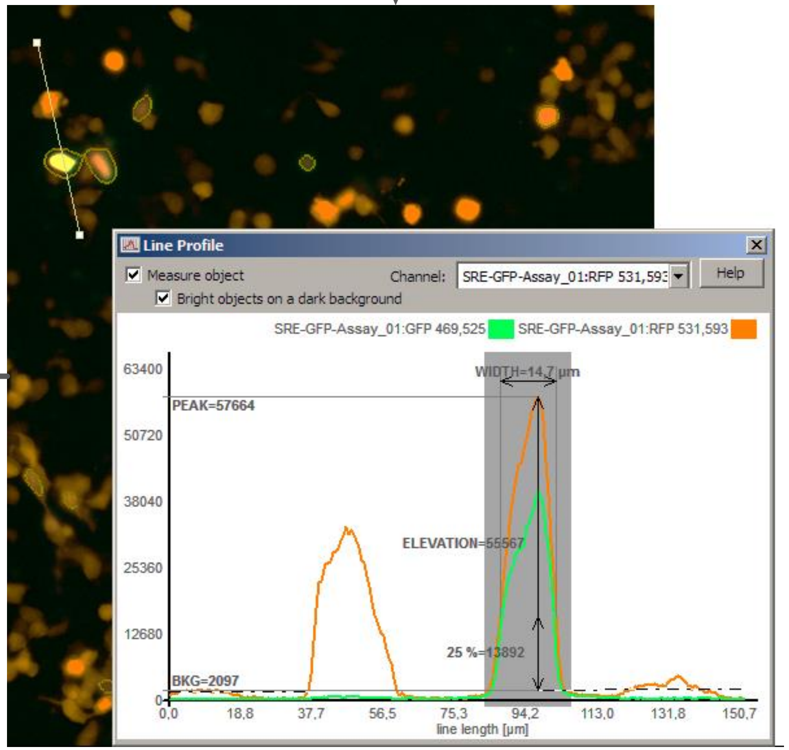

Supplementary methods SM03.

Process of data acquisition for SRF-reporter assay with digital microscopy.

Example 1: Control conditions

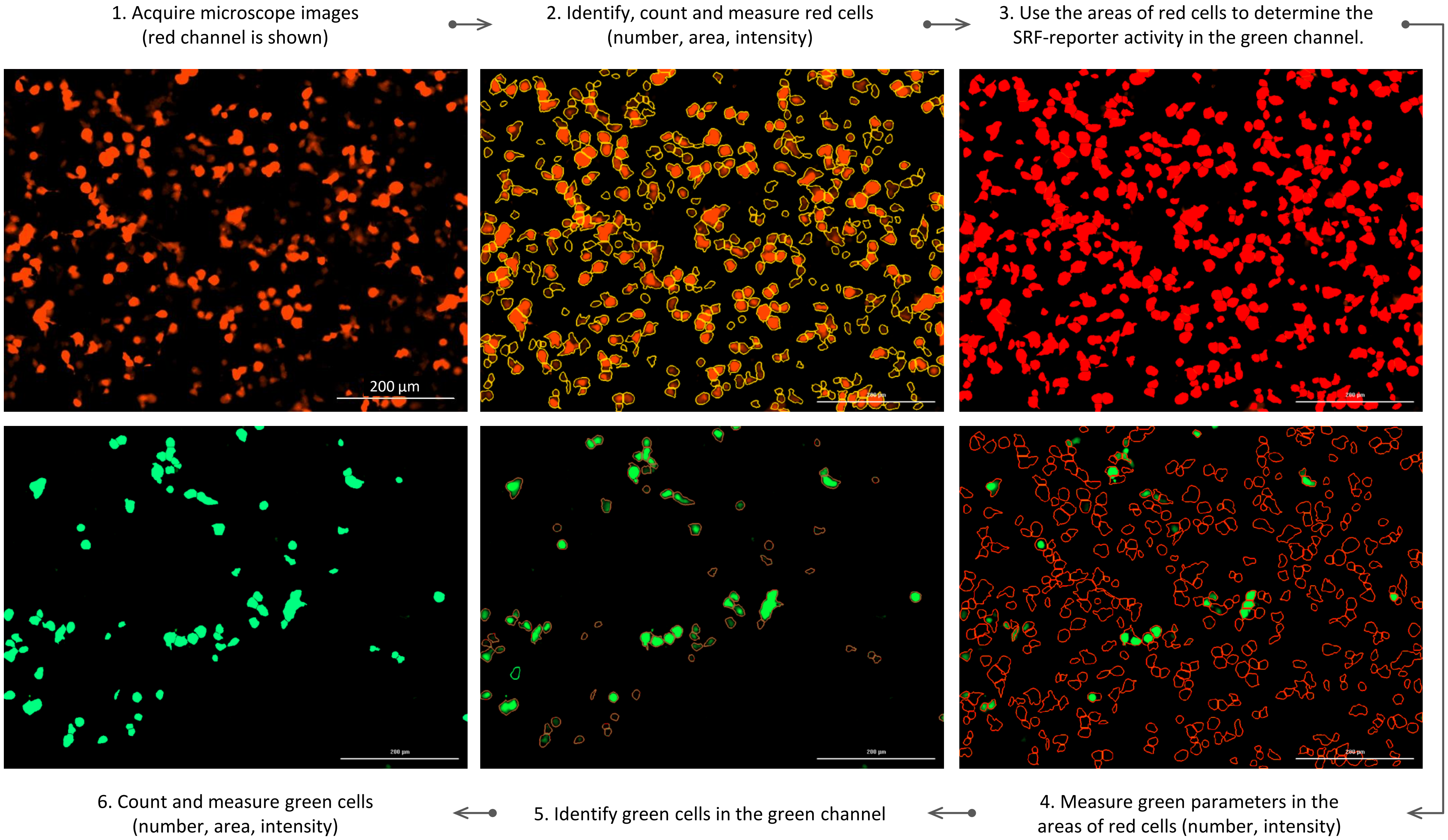

Supplementary methods SM04.

Process of data acquisition for SRF-reporter assay with digital microscopy.

Example 2: 24 h stimulation with 10 µg/l EGF.

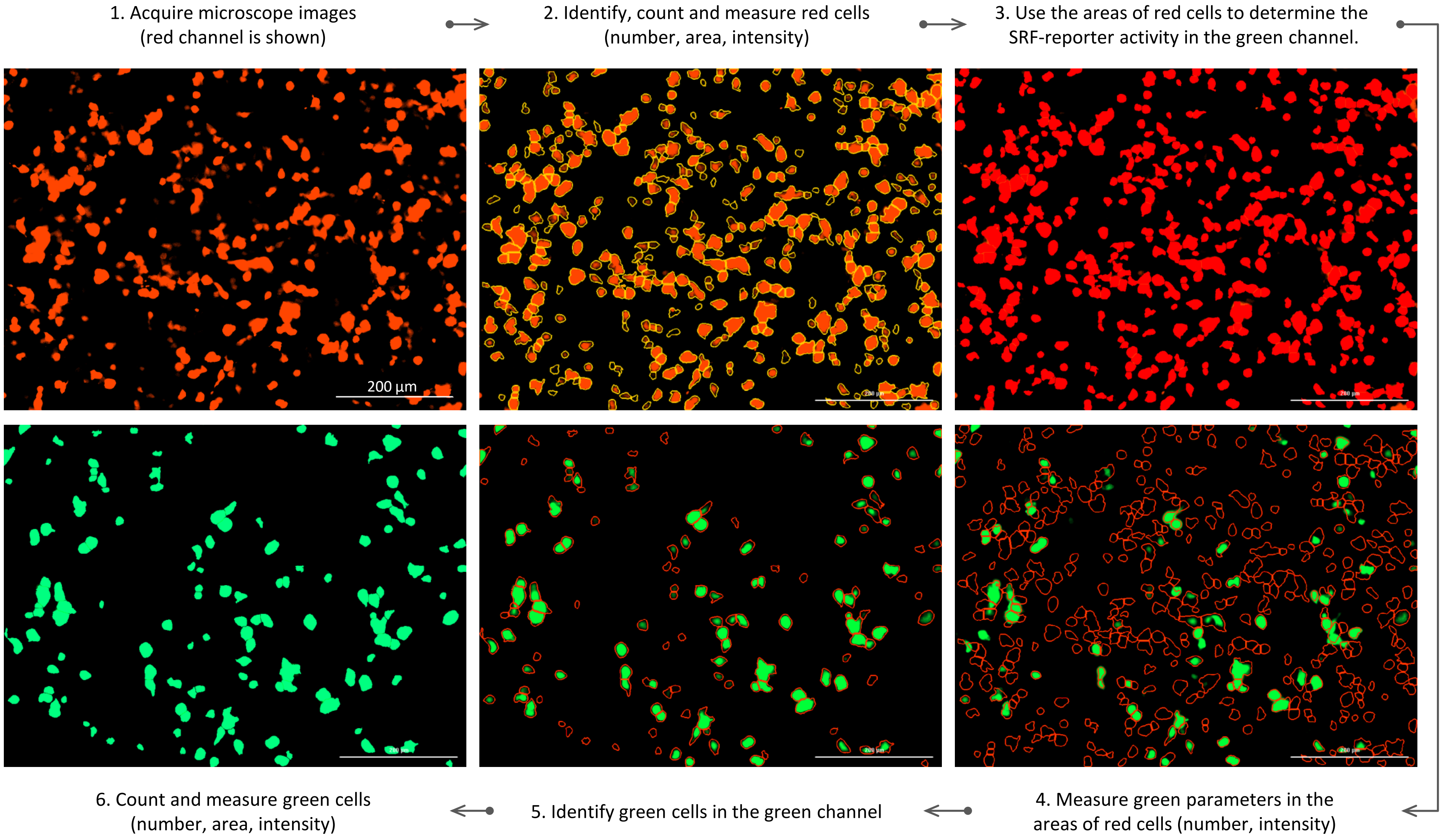

Supplementary methods SM05.

Process of data acquisition for SRF-reporter assay with digital microscopy.

Example 3: 24 h stimulation with 1 µmol/l PMA.

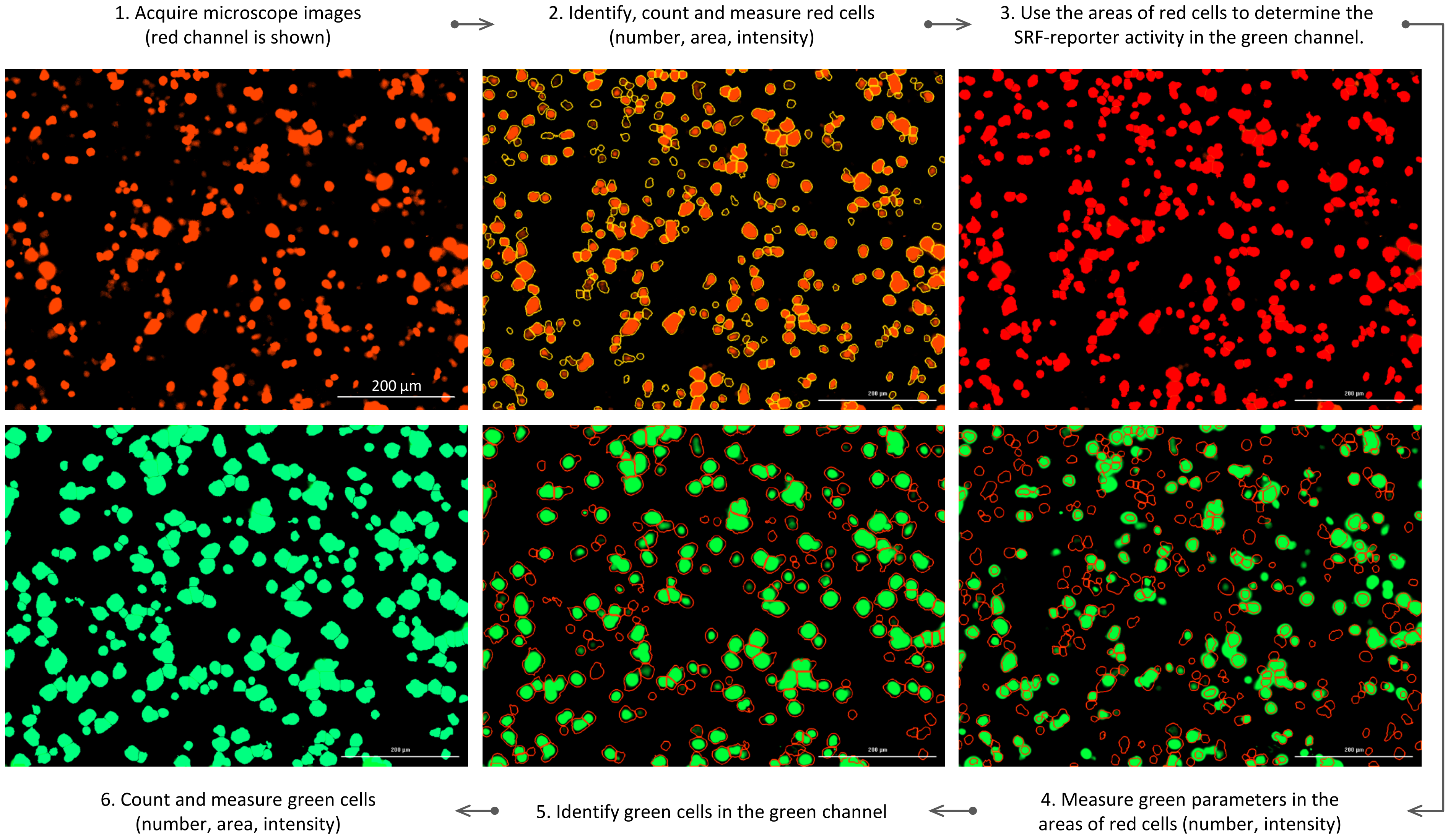

Supplementary methods SM06.

Test comparison of SRF-reporter assay results obtained by single cell digital microscopy or fluorometry.

Columns A-J were incubated with different stimuli for 24 h. Column A = Control.

The upper panel shows an overview of microscopic images of the green channel (= SRF-reporter activity)

The middle panel shows the results of the single cell digital microscopy analysis of the parameters „number of green cells per area“ and mean green fluorescene of transfected cells (=red cells).

The lower panel shows the results of the fluorometry analysis of the parameter „green fluorescence intensity per well“.

A B C D E F G H I J

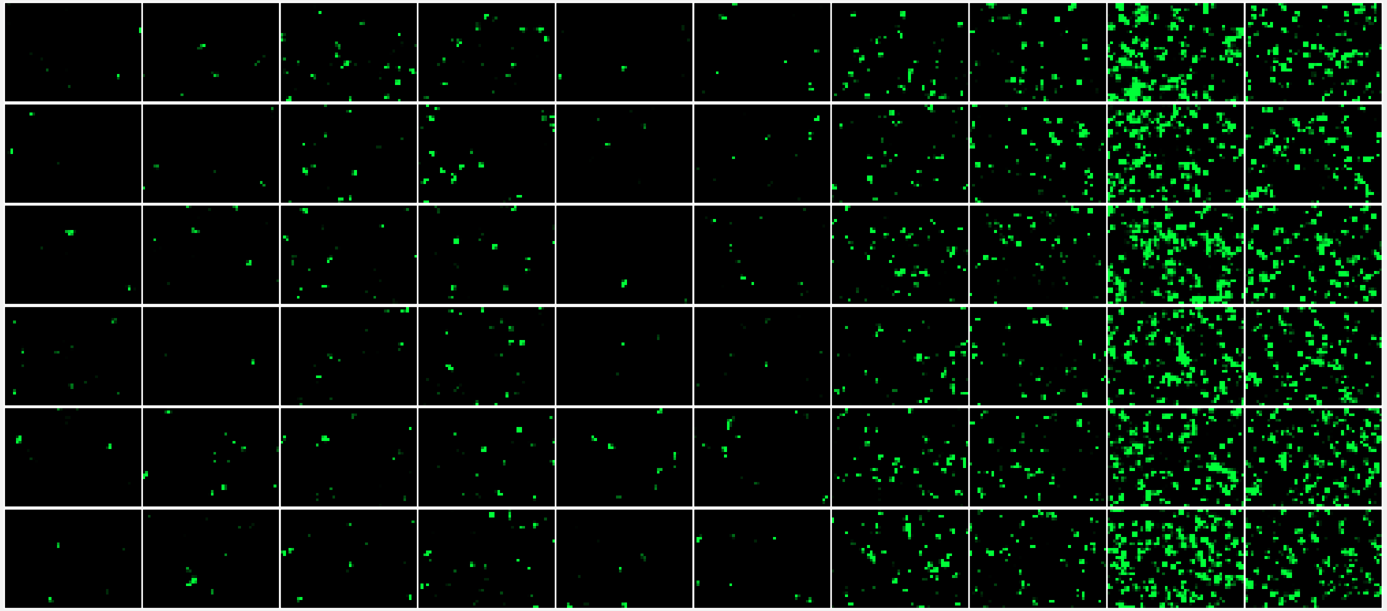

Overview of microscopic images of the green channel

single cell digital microscopy

Number of green cells per area

42	39	82	62	43	38	110	102	211	178
25	25	40	73	41	36	91	110	217	158
31	37	60	60	21	35	132	112	221	166
48	25	61	76	18	43	83	102	210	157
19	35	48	45	40	46	108	103	207	209
27	43	38	80	23	38	117	133	245	189

mean green fluorescene of transfected cells (=red cells)

982	681	2271	2008	818	795	3977	4606	26529	14429
818	672	978	2937	627	898	3009	6097	19635	11789
749	697	1270	1689	633	843	4720	4631	19654	12237
945	444	1044	1921	510	587	3394	3774	19149	13061
963	1064	764	1222	1354	1272	5047	3920	18232	13029
449	716	1012	1875	639	949	6115	4646	22437	10444

fluorometry

green fluorescence intensity per well

525	434	828	567	235	348	842	932	5969	3077
460	384	461	853	208	107	568	1122	4828	2580
393	285	347	508	294	228	865	880	5563	3571
428	198	400	704	279	267	773	680	4341	2904
437	243	280	512	266	301	1017	860	4650	3672
243	231	320	501	260	312	1246	1010	5978	2916

Supplementary methods SM07.

Correlation of the SRF-reporter assay results obtained by single cell digital microscopy or fluorometry from **Supplementary methods SM06**.

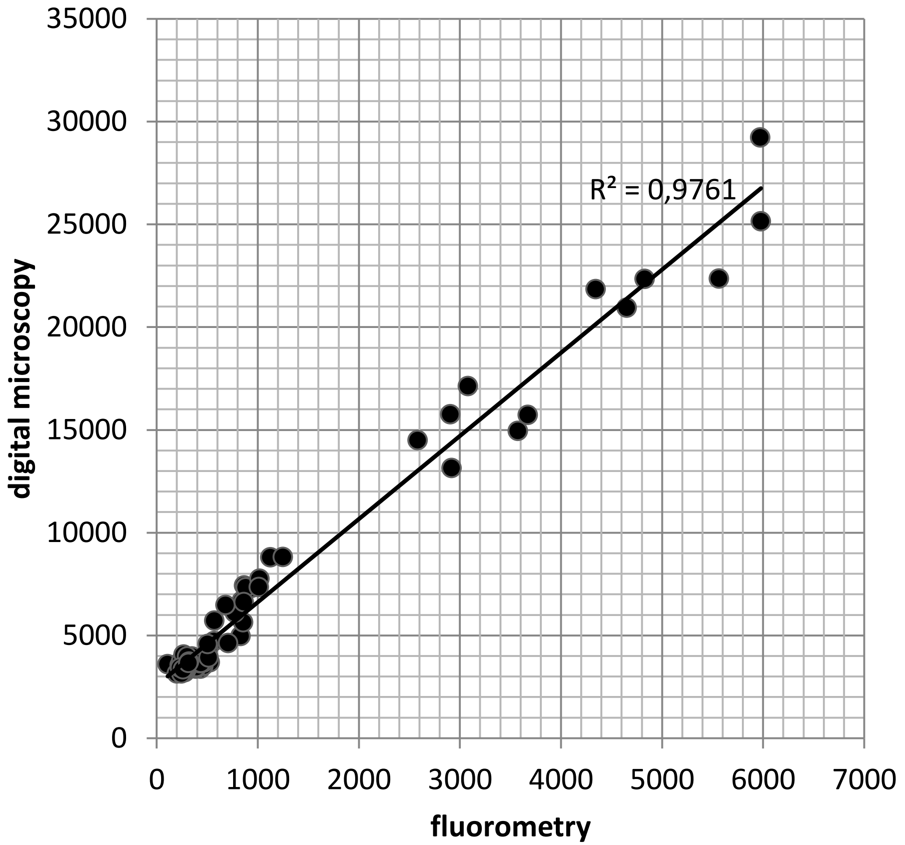

Supplementary methods SM08.

Examples of microscopy images and process of data acquisition for phospho-pERK1/2 in-cell-ELISA.

Cell were transfected with AT1R.

EGFP served as transfection control (green channel).

Phospho-pERK1/2 was detected with a rabbit primary antibody and an AlexaFluor568 labeled secondary antibody (red channel).

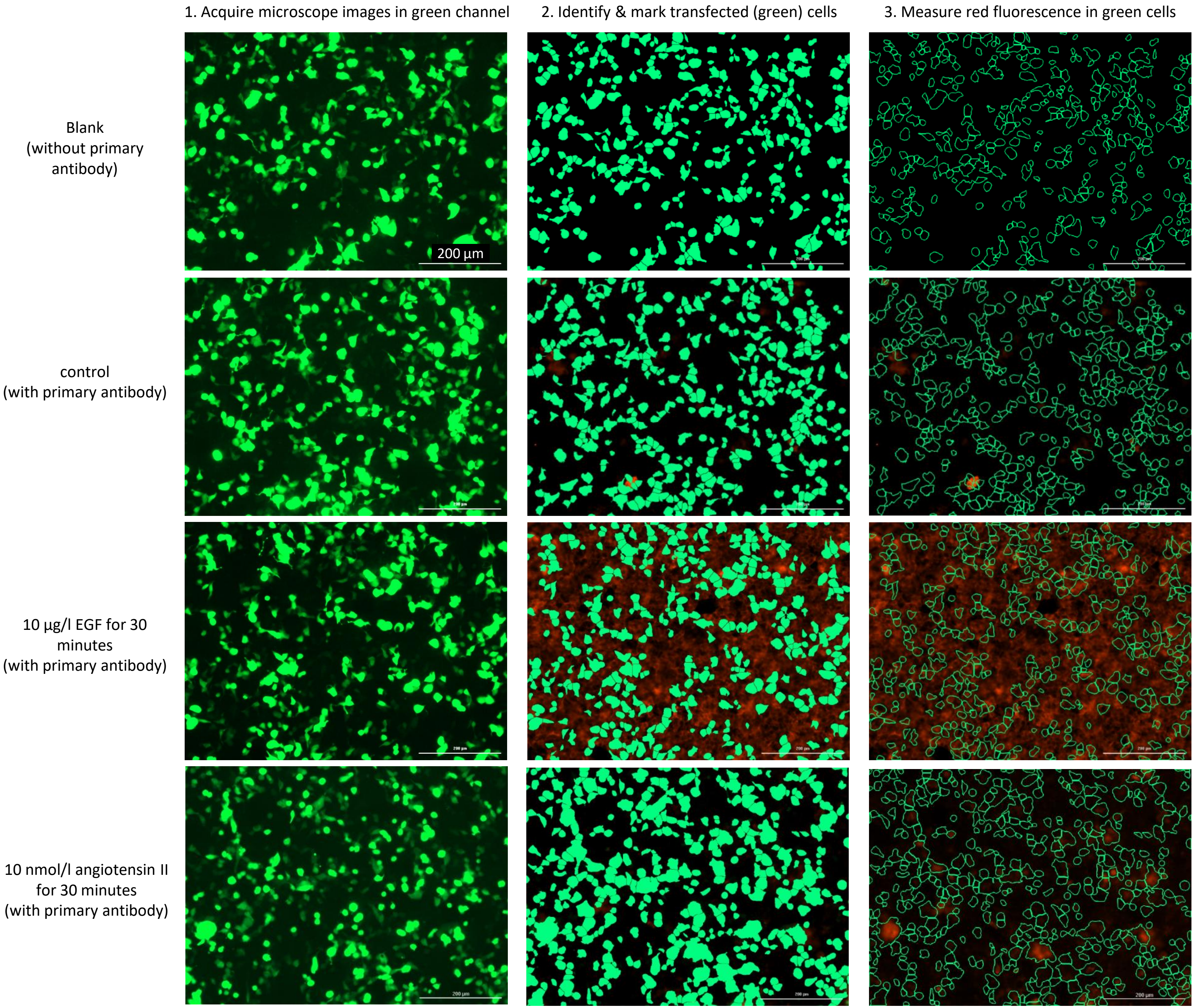

Supplementary methods SM09.

Test comparison of in-cell-ELISA results for ppERK1/2 obtained by single cell digital microscopy or fluorometry.

Columns A-J were incubated with different stimuli for 30 minutes.

Column A = Blank. Column B = control.

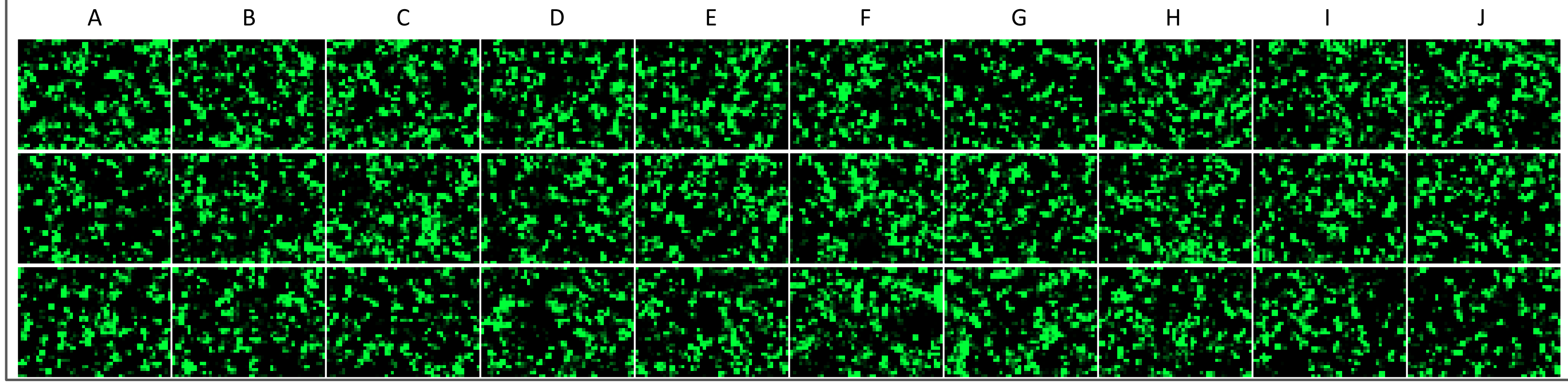

Overview of microscopic images of the green channel

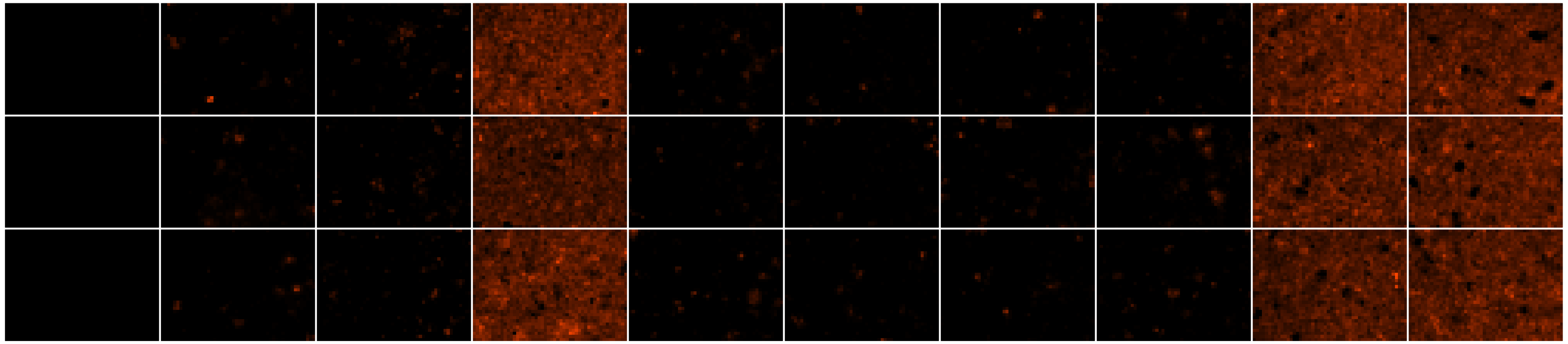

Overview of microscopic images of the red channel (= ppERK1/2)

single cell digital microscopy

mean red fluorescene (= ppERK1/2) of transfected cells

1195	1643	1734	6363	1763	1699	1678	1746	6356	5894
1167	1832	1794	5089	1689	1764	1846	1912	5667	6030
1174	1669	1690	6284	1792	1664	1706	1742	5439	5990

fluorometry

red fluorescence intenstity per well

1216	1715	1877	6414	1828	1781	1762	1772	6611	5952
1173	1873	1928	5167	1744	1850	1925	1974	5889	6160
1182	1679	1918	6563	1835	1733	1751	1716	5637	6118

Supplementary methods SM10. Comparison of pERK1/2-phosphorylation by in-cell-ELISA and single cell digital microscopy in AT1R-WT-transfected and non-transfected cells of one population. Cells were incubated 30 minutes. Histogramms for the distribution of the ppERK1/2-signal (red fluorescence) at the single cell level are shown. The range of the x-axis varies. Quantification see next figure.

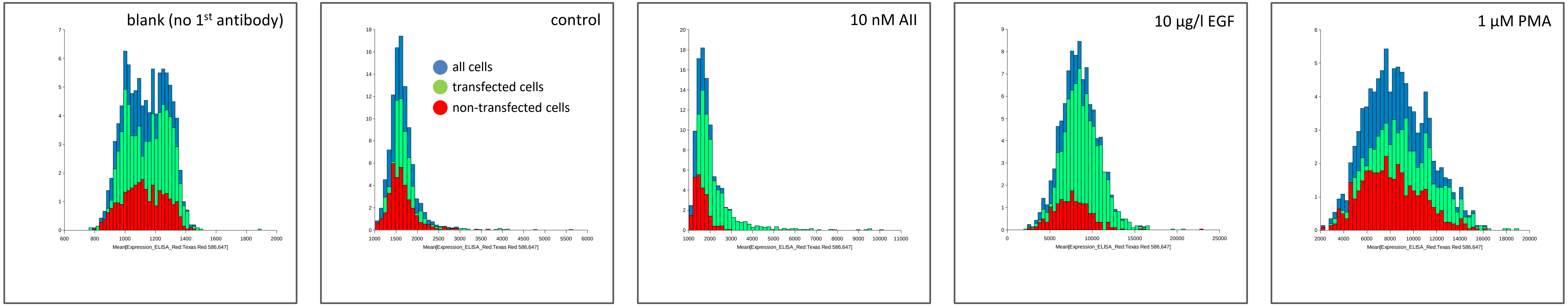

Supplementary methods SM11.

Quantification of pERK1/2-phosphorylation by in-cell-ELISA and single cell digital microscopy in AT1R-WT-transfected and non-transfected cells out of one mixed population.

Cells were incubated for 30 minutes.

The upper tables shows the results for transfected cells (identified by green fluorescence).

The lower tables shows the results for non-transfected cells (identified by the lack of green fluorescence).

The show that only cells transfected with AT1R respond to angiotensin II, whereas all cells respond to EGF and PMA that served as positive controls.

single cell digital microscopy

blank	control	0.1 nM AII	1 nM AII	10 nM AII	10 µg/l EGF	1 µM PMA	
1165	1655	1730	1994	3014	7848	8712	mean red fluorescene (= ppERK1/2) of **transfected** cells
1143	1640	1777	1971	2339	8984	8828	
1326	1660	1742	1998	2328	8699	9828	
**mean**	**100**	**122**	**176**	**306**	**1658**	**1797**	% of control
**sd**	**2**	**5**	**3**	**73**	**110**	**114**	

single cell digital microscopy

1166	1454	1568	1613	1689	6924	8754	mean red fluorescene (= ppERK1/2) of **non-transfected** cells
1189	1644	1491	1580	1600	6849	8218	
1221	1604	1582	1636	1654	7419	8488	
**mean**	**100**	**95**	**111**	**121**	**1564**	**1944**	% of control
**sd**	**22**	**11**	**6**	**10**	**67**	**58**	

Supplementary methods SM12.

Example of in-cell-ELISA for ppERK1/2 in cells transfected with AT1R-WT, obtained by single cell digital microscopy.

Columns A-J were incubated with different stimuli for 30 minutes.

A = blank
B = control
C = 0.1 nM AII
D = 1 nM AII
E = 10 nM AII
F = 10 µg/l EGF
G = EGF + 0.1 AII
H = EGF + 1 AII
I = EGF + 10 AII
J = 1 µM PMA

The data show that EGF, PMA as well as AII induced pERK1/2-phosphorylation and that the effects of AII and EGF are not additive.

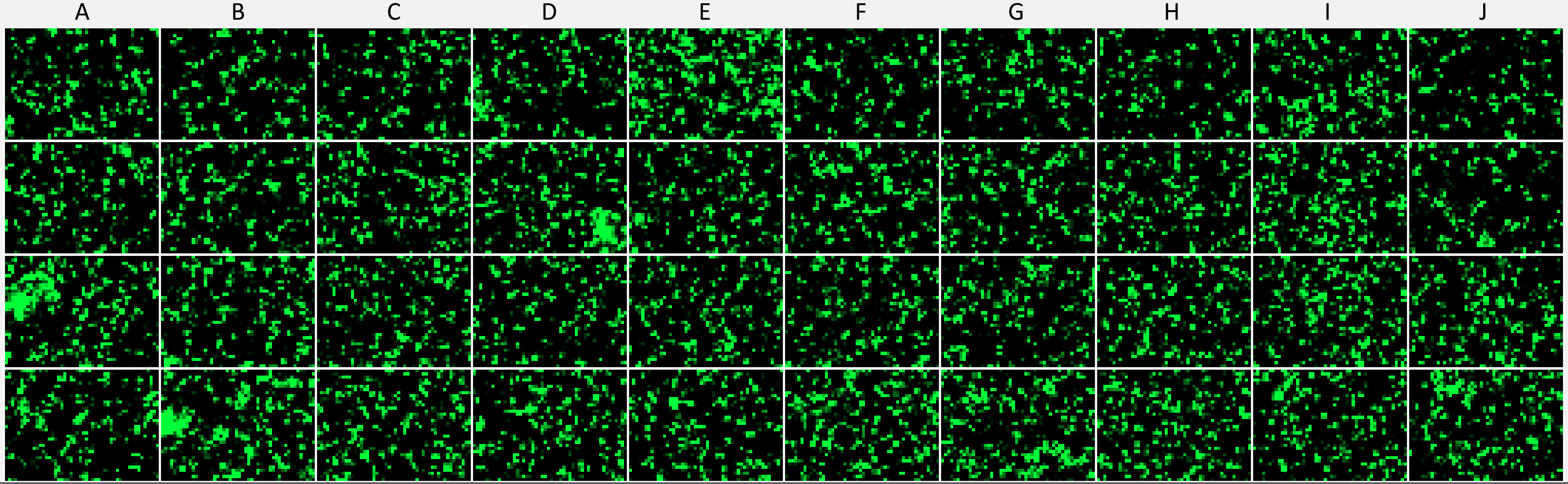

Overview of microscopic images of the green channel (= transfected cells)

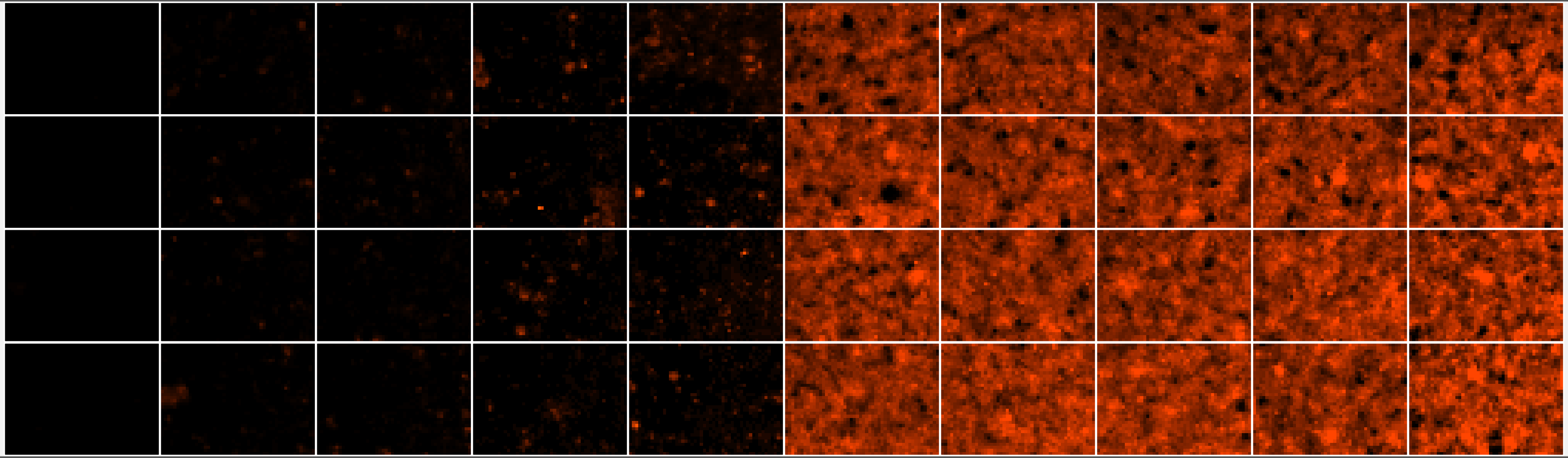

Overview of microscopic images of the red channel (ppERK1/2)

single cell digital microscopy

mean red fluorescene (= ppERK1/2) of transfected cells

1171	1689	1721	2028	2977	8056	8260	8057	8106	8736
1147	1683	1785	2094	2332	9189	9004	9188	9712	9478
1345	1692	1761	1994	2349	8923	9204	9408	10319	9725
1184	1754	1803	1910	2228	8826	9799	10044	9673	10529
**mean**	**100**	**111**	**159**	**263**	**1578**	**1628**	**1608**	**1721**	**1644**
**sd**	**2**	**8**	**22**	**92**	**81**	**105**	**172**	**189**	**136**

% of control

Supplementary methods figure SM13.

Example of in-cell-ELISA for cFOS in cells transfected with AT1R, obtained by single cell digital microscopy.

Cells were transfected with AT1R-WT.

Columns A-G were incubated with different stimuli for 6 h.

A = Blank

B = control

C = 1 nM AII

D = 10 nM AII

E = 10 µg/l EGF

F = EGF + 1 nM AII

G = EGF + 10 nM AII.

The data show (i) that EGF induces cFOS-expression independently of AT1R expression, whereas AII exerts no effect in transfected or non-transfected cells. Furthermore, the data show (ii) a synergistic effect of EGF and AII on cFOS-expression in AT1R-transfcted cells (compare column E with columns F and G in the upper table) but not in non-transfected cells (lower table).

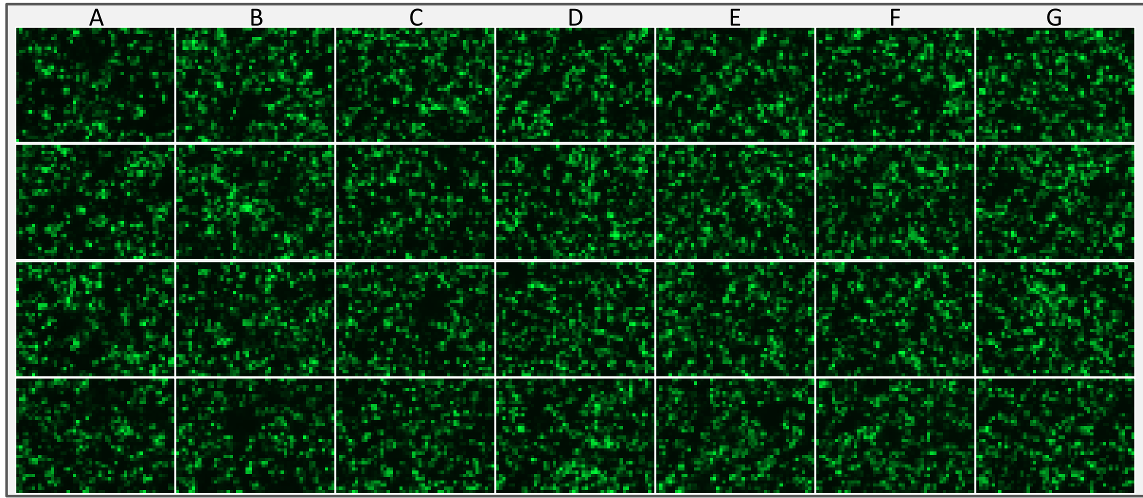

Overview of microscopic images of the green channel (= transfected cells)

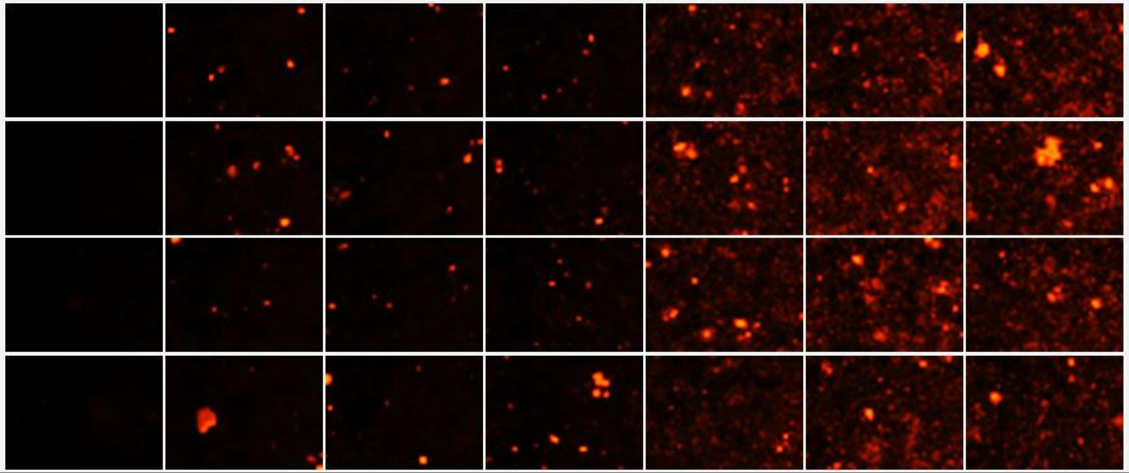

Overview of microscopic images of the red channel (= cFOS)

single cell digital microscopy

mean red fluorescene (= cFOS) of **transfected** cells

1123	1373	1661	1636	2762	3195	3419
1084	1567	1666	1792	2575	3250	3635
1103	1689	1609	1736	2395	3112	3355
1123	1538	1713	1855	2421	2937	3255
**mean**	**100**	**128**	**149**	**330**	**465**	**532**
**sd**	**26**	**8**	**19**	**34**	**27**	**32**

% of control

single cell digital microscopy

mean red fluorescene (= cFOS) of **non-transfected** cells

1014	1527	1425	1488	2592	2463	2594
1051	1540	1545	1425	2550	2636	2548
1062	1463	1464	1420	2581	2690	2573
1062	1480	1457	1467	2426	2657	2424
**mean**	**100**	**93**	**88**	**327**	**344**	**327**
**sd**	**7**	**10**	**6**	**15**	**19**	**14**

% of control

Supplementary methods SM14.

Comparison of cFOS-expression by in-cell-ELISA and single cell digital microscopy in AT1R-WT-transfected and non-transfected cells out of one mixed population, at the individual cell level. Histogramms for the distribution of the cFOS-signal for transfected and non-transfected cells at the single cell level are shown. For quantification see **supplementary methods SM13**.

Cells were incubated for 6 h.

EGF leads to an increased cFOS expression of transfected and non-tranfected cells with a similar intensity profile (see middle panel). The addition of angiotensin II (AII) leads to an additional increase of cFOS-expression only in transfected (= AT1R expressing) cells (see the different expression profile of transfected and non-transfected cells in the lower panel).

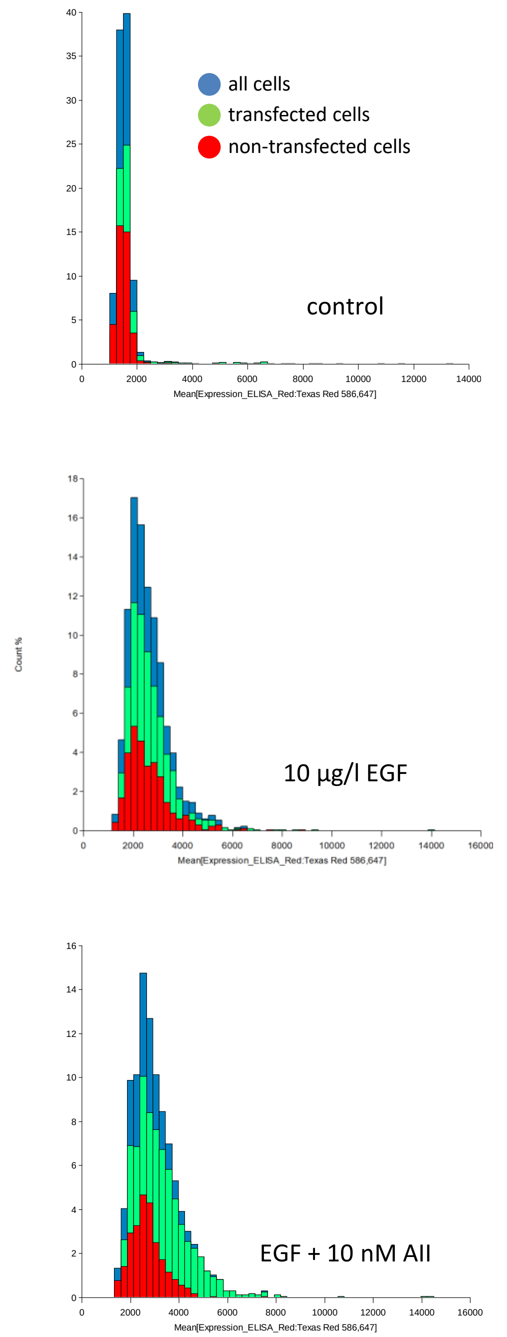

Supplementary methods SM15. Confirmation immunoblots of EGF-AII-synergism concerning cFOS-expression in cells expressing AT1R-WT. The four experiments presented here confirm the data obtained by in-cell-ELISA.

c-Fos immunoblot
HEK-cells transfected with AT1R-WT
experiments 1 and 2

Incubation: 6 h
Lane 1+2: control
Lane 3+4: 10 µg/l EGF
Lane 5+6: 10 nM angiotensin II
Lane 7+8: angiotensin II + EGF

Antibodies:
1. ab: c-Fos (CST) 1:1000
2. ab: anti-rabbit-HRP (CST) 1:1000

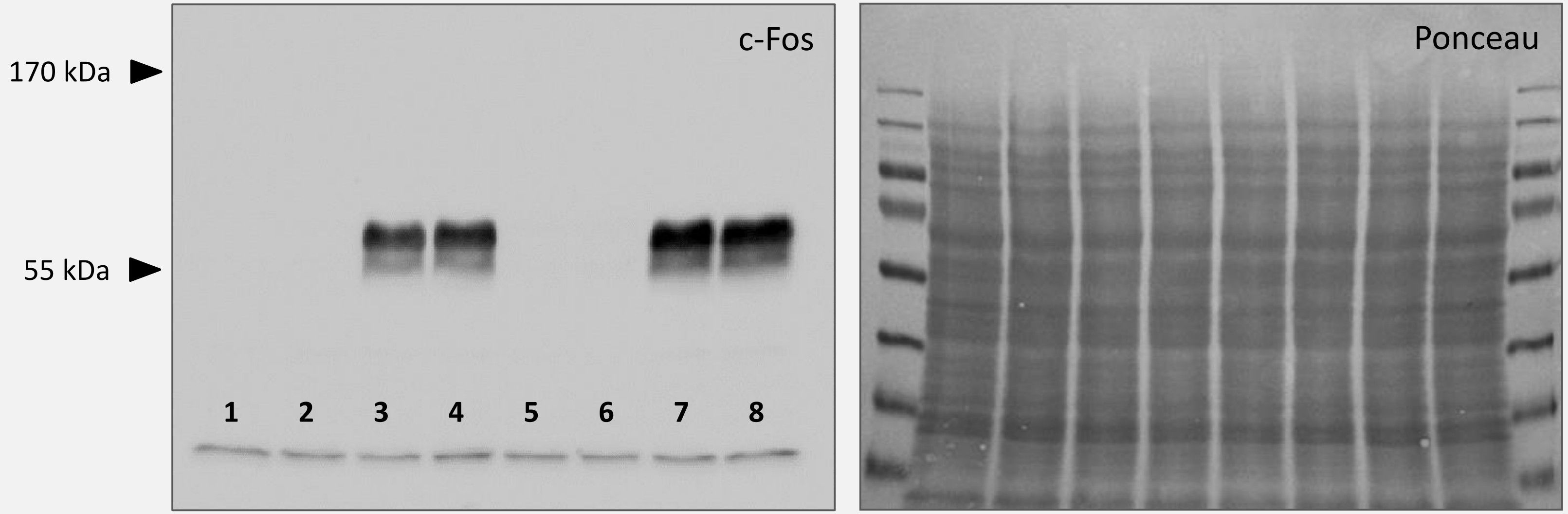

c-Fos immunoblot
HEK-cells transfected with AT1R-WT
experiments 3 and 4

Incubation: 6 h
Lane 1+2: control
Lane 3+4: 10 µg/l EGF
Lane 5+6: 10 nM angiotensin II
Lane 7+8: angiotensin II + EGF

Antibodies:
1. ab: c-Fos (CST) 1:1000
2. ab: anti-rabbit-HRP (CST) 1:1000

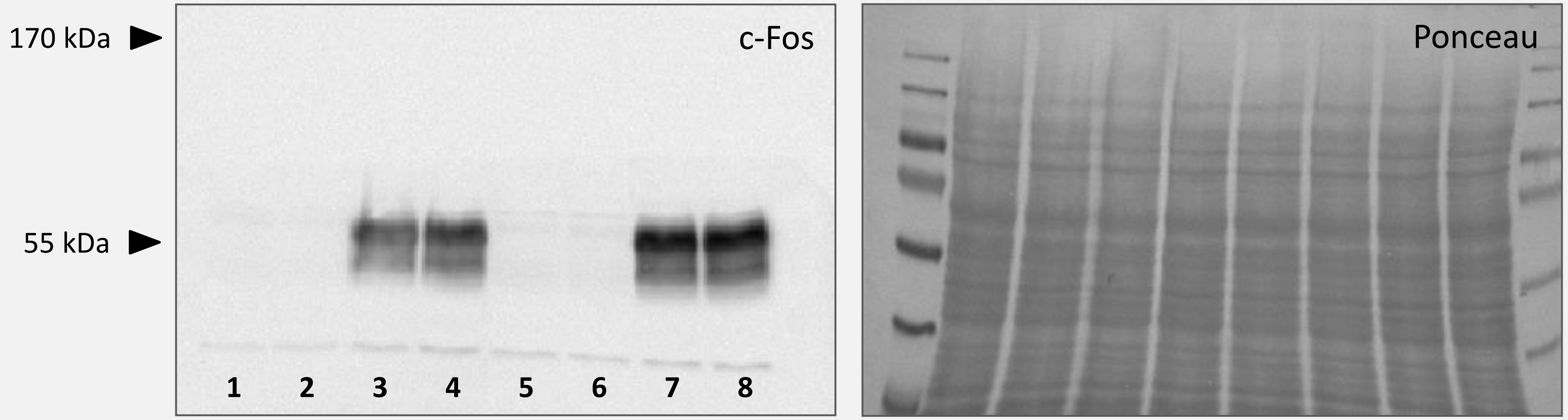

Supplementary methods SM16. Confirmation immunoblots for EGF-AII-synergism concerning cFOS-expression in cells expressing AT1R-MUT1. The four experiments presented here confirm the data obtained by in-cell-ELISA.

c-Fos immunoblot
HEK-cells transfected with AT1R-MUT
experiments 1 and 2

Inkubation: 6 h
Lane 1+2: control
Lane 3+4: 10 µg/l EGF
Lane 5+6: 10 nM angiotensin II
Lane 7+8: angiotensin II + EGF

Antibodies:
1. ab: c-Fos (CST) 1:1000
2. ab: anti-rabbit-HRP (CST) 1:1000

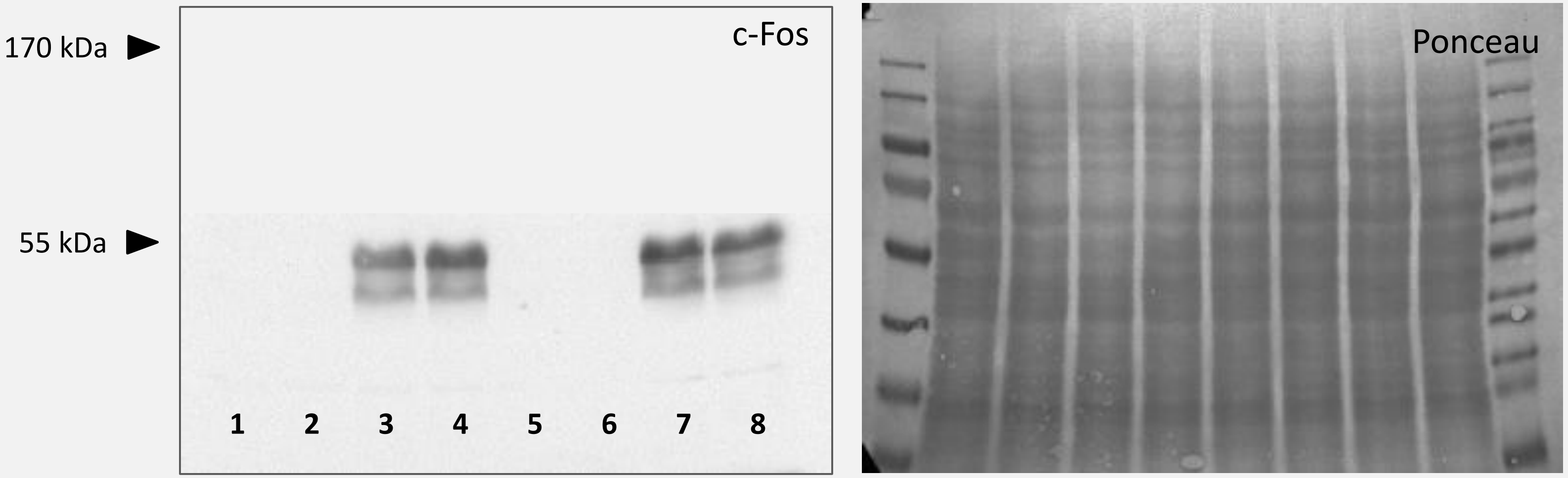

c-Fos immunoblot
HEK-cells transfected with AT1R-MUT
experiments 1 and 2

Inkubation: 6 h
Lane 1+2: control
Lane 3+4: 10 µg/l EGF
Lane 5+6: angiotensin II + EGF
Lane 7+8: 10 nM angiotensin II

Antibodies:
1. ab: c-Fos (CST) 1:1000
2. ab: anti-rabbit-HRP (CST) 1:1000

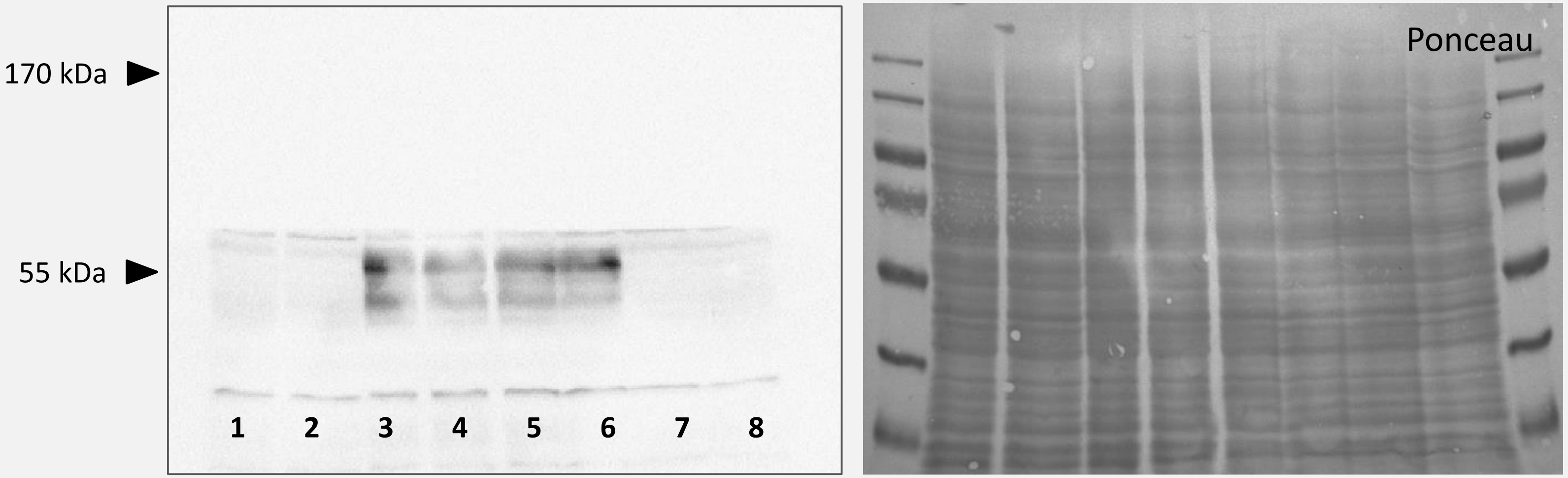

Supplementary methods SM17. Analysis of the immunoblot results for EGF-AII-synergism concerning cFOS-expression in cells expressing AT1R-WT or AT1R-MUT1. The data here confirm the data obtained by in-cell-ELISA. Statistical analysis was performed by ANOVA. (N/n = 4/4).

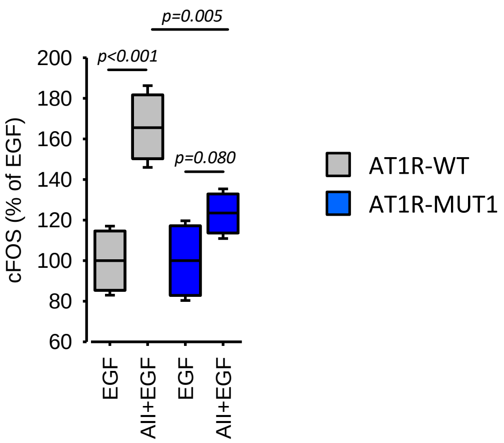

Supplement: Supplementary file 2 — Supplementary file2 (PDF 4285 KB) [file 18_2024_5281_MOESM2_ESM.pdf]
